# Al Nanoparticle‐Decorated Metal Oxide Synaptic Transistors for Ultralow‐Energy Neuromorphic Computing with Wide Dynamic Range

**DOI:** 10.1002/advs.202521321

**Published:** 2025-11-30

**Authors:** Jun‐Gyu Choi, Yoonseok Song, Seokhyeon Baek, Jingon Jang, Sungjun Park

**Affiliations:** ^1^ Department of Electrical and Computer Engineering Ajou University Suwon 16499 Republic of Korea; ^2^ Department of Intelligence Semiconductor Engineering Ajou University Suwon 16499 Republic of Korea; ^3^ School of Computer and Information Engineering Kwangwoon University Seoul 01897 Republic of Korea

**Keywords:** low‐energy consumption, neuromorphic device, synaptic plasticity

## Abstract

Achieving ultralow energy consumption alongside high synaptic fidelity remains a key challenge in the development of practical and scalable neuromorphic hardware systems. Electrolyte‐gated memtransistors (EGMTs), which enable low‐voltage analog switching via electric double layer modulation, suffer from a fundamental trade‐off between dynamic range and energy consumption. Here, a nanoparticle‐engineered EGMT is reported that mitigates this limitation by incorporating aluminum nanoparticles at the interface between a solution‐processed indium gallium zinc oxide channel and a solid polymer electrolyte composed of polyethylene oxide doped with lithium hexafluoroarsenate. This design yields 50 discrete conductance states at a drain voltage of 1 mV, achieving a dynamic range exceeding 78 and a synaptic switching energy of 0.62 pJ spike^−1^, which ranks among the lowest reported for EGMTs. Neural network simulations (784  ×  60  ×  10), based on experimentally extracted conductance updates, predict energy savings of 99.7% during training and 91.4% during inference compared to digital complementary metal–oxide–semiconductor implementations.

## Graphical abstract

A nanoparticle‐engineered electrolyte‐gated memtransistor is introduced as a materials‐level strategy to overcome the intrinsic trade‐off between energy consumption and synaptic precision. By embedding aluminum nanoparticles at the oxide–electrolyte interface to modulate ion trapping dynamics, the device achieves stable multistate plasticity under millivolt operation, enabling a practical route toward scalable ultralow‐energy neuromorphic hardware.

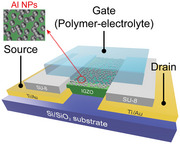

## 1 Introduction

Energy efficiency is a core requirement for the practical deployment and commercialization of neuromorphic computing systems, where the achievable system‐level efficiency is ultimately constrained by the switching energy consumption of individual synaptic devices.^[^ ^1^, ^2^, ^3^, ^4^ ^]^ Inspired by the massively parallel information processing of the human brain, neuromorphic architectures offer efficient handling of big data and overcome the memory bottlenecks and high energy consumption inherent to conventional complementary metal–oxide–semiconductor (CMOS)‐based von Neumann architectures.^[^ ^5^, ^6^, ^7^ ^]^ Beyond energy efficiency, finer modulation of synaptic weights is essential for the more accurate representation of analog signals during the learning and inference stages.^[^ ^8^, ^9^ ^]^ From a device‐level perspective, as integration density and circuit complexity increase, minimizing the voltage burden on individual devices is essential to reduce cumulative energy consumption over time,^[^ ^10^ ^]^ while ensuring precise control of synaptic weights across a wide dynamic range (*DR*).^[^ ^11^ ^]^ To ensure reliable and stable synaptic behavior with a wide *DR* under low‐voltage operation, strong local electric fields across the dielectrics must be maintained at the device level. However, typical memristive devices face fundamental material constraints, such as limits on dielectric constants and leakage currents due to quantum tunneling at reduced dielectric thicknesses, which generally necessitate operating voltages above 1 V.^[^ ^12^, ^13^, ^14^ ^]^

Electrolyte‐gated memtransistors (EGMTs) have emerged as promising candidates for energy‐efficient neuromorphic computing.^[^ ^15^, ^16^ ^]^ Their unique electrostatic gating mechanism, enabled by sub‐nanoscale thicknesses of electric double layers (EDLs), yields exceptional gating field effects even under sub‐1 V operation, owing to their high areal capacitance.^[^ ^17^, ^18^ ^]^ This facilitates continuous, analog modulation of channel conductance for gradual synaptic weight updates. Metal–oxide–semiconductors used as EGMT channels offer additional advantages, including intrinsic amplification capability for achieving a wide *DR*, along with environmental stability, scalability, and compatibility with typical circuit integration.^[^ ^19^, ^20^ ^]^ These aspects offer a strong potential for EGMTs to realize low‐power, high‐precision, and scalable neuromorphic systems.

State‐of‐the‐art EGMTs have demonstrated picojoule‐level energy consumption per synaptic functionality event, of which figure‐of‐merit parameters are summarized in Table S1 (Supporting Information).^[^ ^10^, ^18^, ^19^, ^20^, ^21^, ^22^, ^23^, ^24^, ^25^, ^26^, ^27^, ^28^, ^29^, ^30^, ^31^, ^32^, ^33^, ^34^, ^35^, ^36^, ^37^, ^38^, ^39^, ^40^, ^41^, ^42^, ^43^, ^44^, ^45^, ^46^ ^]^ Through strategic engineering of materials and device designs, such as ion migration‐induced phase transformations^[^ ^21^ ^]^ or dual‐electrolyte‐assisted ionic modulation,^[^ ^22^ ^]^ the energy consumption of EGMTs has reached ≈5 pJ spike^−1^ at 50 mV of drain voltage (*V*_DS_), a critical parameter in synaptic energy calculation.^[^ ^10^ ^]^ Meanwhile, EGMTs have demonstrated an expanded *DR* up to ≈50, while consuming a few tens of picojoules per spike for sign language translation.^[^ ^23^ ^]^

However, the simultaneous realization of ultralow energy consumption and a wide *DR* in EGMTs remains fundamentally limited by the competing requirements of device operation. Minimizing energy consumption typically involves lowering *V*_DS_, which can diminish the conductance modulation sensitivity near the threshold voltage (*V*_TH_), thereby narrowing the effective *DR*. Conversely, expanding the *DR* requires high conductance tunability and a low off‐state current, which often necessitates stronger driving conditions that increase energy usage. Because *DR*, defined as *G*_max_/*G*_min_, determines the resolution of synaptic weights, this tradeoff directly affects learning accuracy and scalability in neuromorphic systems. Overcoming this limitation necessitates a device strategy that carefully balances the energy consumption with the conductance precision under low‐voltage operation.

In this study, we addressed this trade‐off by incorporating nanoparticles (NPs) at the channel surface of an EGMT to achieve both ultralow energy consumption and wide *DR*. The device comprises a solution‐processed IGZO channel and a polyethylene oxide (PEO):LiAsF_6_ electrolyte, with aluminum (Al) NPs positioned at the channel–electrolyte interface to serve as ion trap sites. The critical thickness of Al NPs enlarges the hysteresis window in the transfer curve of EGMTs, enabling effective long‐term synaptic plasticity (LTSP) with stable multilevel conductance modulation at extremely low *V*_DS_ down to 1 mV. In addition, the steady‐state conductance level is lowered to achieve a wide *DR*. The NP‐decorated IGZO EGMT achieves a wide *DR* of over 78 across 50 conductance states, and its energy consumption for a single spike is as low as 0.62 pJ. Compared to digital CMOS implementation, system‐level simulations using a neural network architecture revealed energy reductions of ≈99.7% during training and ≈91.4% during the inference stage. These results highlight the potential of the proposed EGMT as a scalable synaptic device enabling energy‐efficient neuromorphic computing systems.

## 2 Results and Discussion

### 2.1 Design and Electrical Characteristics of NP‐Decorated EGMTs

**Figure 1:**
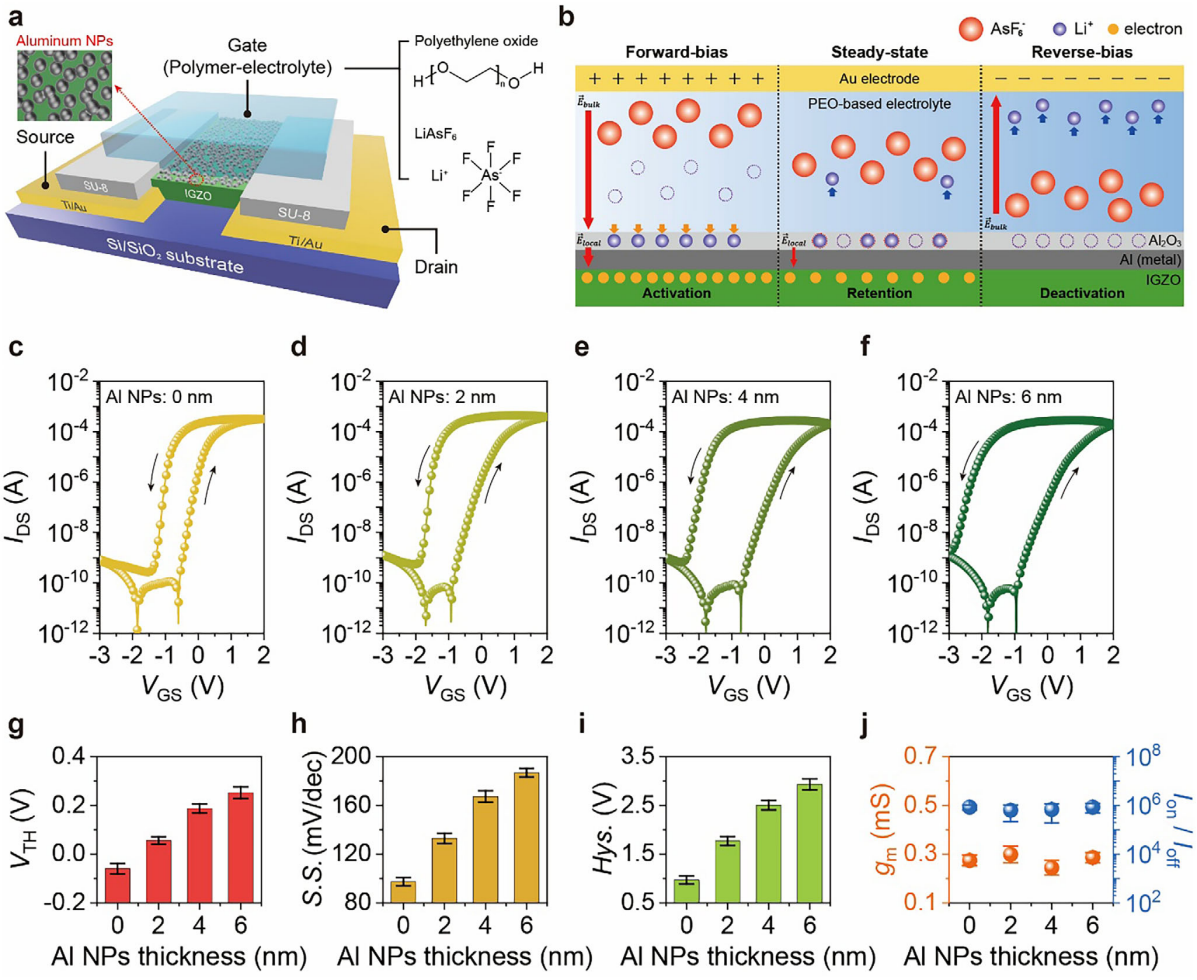
Design and electrical characteristics of EGMTs fabricated with Al‐NP‐decorated IGZO films. a) Schematic of EGMT structure and layered components. b) Schematic of Li^+^ dynamics and field modulation in EGMT with redox‐based trapping/de‐trapping in Al_2_O_3_ under various bias conditions. Representative transfer curves of the EGMTs, where the thicknesses of the Al NPs were c) 0 nm, d) 2 nm, e) 4 nm, and f) 6 nm, under 100 mV *V*_DS_. The nominal NP thicknesses were defined by quartz crystal microbalance monitoring during evaporation. NP thickness‐dependent figure‐of‐merit parameters of EGMT, including g) threshold voltage (*V*_TH_), h) subthreshold swing (*S.S*), and i) hysteresis windows (*Hys*.), j) transconductance (*g*_m_), and on/off current ratio (*I*_on_/*I*_off_), while the error bars denote standard deviations over ten devices.

Within the schematic device structure (**Figure** **1**a), the solution‐processed IGZO film serving as the channel layer of the EGMT offers large‐area processability, superior electrical performance, and robust electrochemical and environmental stability.^[^ ^47^, ^48^, ^49^ ^]^ These advantages contrast with recently reported low‐dimensional material‐based synaptic devices with various ion regulation mechanisms. While such emerging systems demonstrate distinctive functionalities, oxide‐based EGMTs remain highly competitive in terms of environmental stability, scalability, and compatibility with large‐area integration. To minimize leakage current, all channel regions were electrically isolated via conventional photolithography prior to the deposition of an SU‐8 passivation layer. Following the thermal evaporation of Al NPs with specific thicknesses, an electrolyte containing Li^+^‐based salts and PEO was deposited onto the NP‐decorated IGZO surface to formulate an EDL that demonstrated a high areal capacitance for widening *DR* even at low gating loads. Gold (Au) tip electrodes were placed in direct contact with the electrolyte to apply the gate bias.

Considering the effect of material design on EGMT performance,^[^ ^21^, ^22^ ^]^ the EGMT electrolyte was strategically chosen by systematic screening. PEO serves as a matrix for the solid electrolyte because of its superior mechanical and electrochemical stability at the electrolyte–semiconductor interface by limiting the parasitic reactions that are common in liquid systems.^[^ ^50^ ^]^ The solid electrolyte effectively suppressed ion migration‐induced leakage currents and ensured stable ionic gating, making it suitable for neuromorphic applications with reliable operation during repeated synaptic switching cycles.^[^ ^51^ ^]^ In addition to the effects of the polymer matrix, the choice of Li^+^‐based salts substantially influences ionic mobility and relaxation dynamics within the electrolyte.^[^ ^52^ ^]^ Among the salts investigated, the PEO:LiAsF_6_ electrolyte exhibited the widest hysteresis window and the lowest ion mobility, as confirmed by electrical comparisons with LiBF_4_, LiClO_4_, and LiPF_6_ systems (Figures S1–S4, Supporting Information). This performance is attributed to the large ionic radius of AsF_6_^−^, which further impedes Li^+^ migration under applied gate bias, effectively extending ion relaxation times and enhancing memory retention.

Despite these material‐level improvements, conventional electrolyte‐gated transistors often suffer from insufficient long‐term synaptic plasticity (LTSP), primarily due to the transient and weak interactions between mobile ions and the channel surface.^[^ ^19^, ^24^ ^]^ To address this limitation, Al NPs were strategically introduced at the electrolyte–IGZO interface. A conceptual schematic depicts the dynamics of mobile Li^+^ under different pulse conditions (Figure 1b). Following thermal evaporation, the Al NPs readily form a native oxide layer (Al_2_O_3_) upon exposure to ambient air, providing abundant trapping sites for field‐driven Li^+^.^[^ ^52^ ^]^ The small ionic radius of Li^+^ facilitated semi‐reversible electrochemical trapping within the interstitial or substitutional sites of Al_2_O_3_ via Faradaic redox reactions.^[^ ^52^, ^53^ ^]^ By pinned Li^+^ within the Al_2_O_3_ shell, a localized electric field accumulated electrons in the IGZO channel even after the removal of the forward‐bias pulse. The trapped Li^+^ were then removed using a reverse bias pulse, eliminating the local field and deactivating the channel.

To evaluate the effects of the NPs in enhancing synaptic function behavior, their nominal thickness on the IGZO surface was systematically varied from 0 to 6 nm. Each NP thickness corresponds to the value monitored in situ using a quartz crystal microbalance during thermal evaporation. All EGMTs exhibited typical n‐type switching behavior with a high on/off current ratio (*I*_on_/*I*_off_) of over ≈10^6^ owing to EDL‐driven amplification and featured counterclockwise hysteresis loops under a constant gate voltage (*V*_GS_) range from −3 to 2 V, for which *V*_DS_ was fixed at 100 mV (Figure 1c–f). Notably, increasing NP thickness resulted in a progressive enlargement of the hysteresis window, attributed to enhanced electrochemical trapping of Li⁺ ions. For quantitative analysis, the figure‐of‐merit parameters of the corresponding EGMTs, including *V*_TH_, subthreshold swing (*S.S*.), and hysteresis window size (*Hys*.), *g*_m_, and *I*_on_/*I*_off_ were statistically evaluated by measuring ten devices (Figure 1g–j). Interestingly, an increase in the NP thickness resulted in a positive *V*_TH_ shift during forward sweeping. When a positive field is applied, the mobile Li^+^ are driven toward the NP‐decorated IGZO surface. This shift is attributed to the partial obstruction of Li⁺ access to the IGZO surface by the NPs, effectively increasing the ion–channel distance and requiring a stronger electric field to form the conduction channel. Furthermore, both *S.S*. and hysteresis window width increased with thicker NP layers, indicating that denser NPs introduce more persistent trap sites at the interface. Notably, despite the enhanced trap effects, the devices demonstrated consistent *g*_m_ and *I*_on_/*I*_off_ levels, indicating no significant change in the EDL capacitance with an increase in the NP thickness up to 6 nm. This observation suggests that once the applied *V*_GS_ surpasses the increased *V*_TH_, a stable conduction channel is established, and the subsequent charge transport is minimally influenced by the NPs. Importantly, although *V*_TH_ shifted positively, the NP‐decorated EGMTs retained low‐voltage operation even below 1 V *V*_DS_, highlighting their continued suitability for neuromorphic applications that require ultralow energy consumption.

### 2.2 Interfacial Characterizations of Al NP‐Decorated IGZO Films

**Figure 2:**
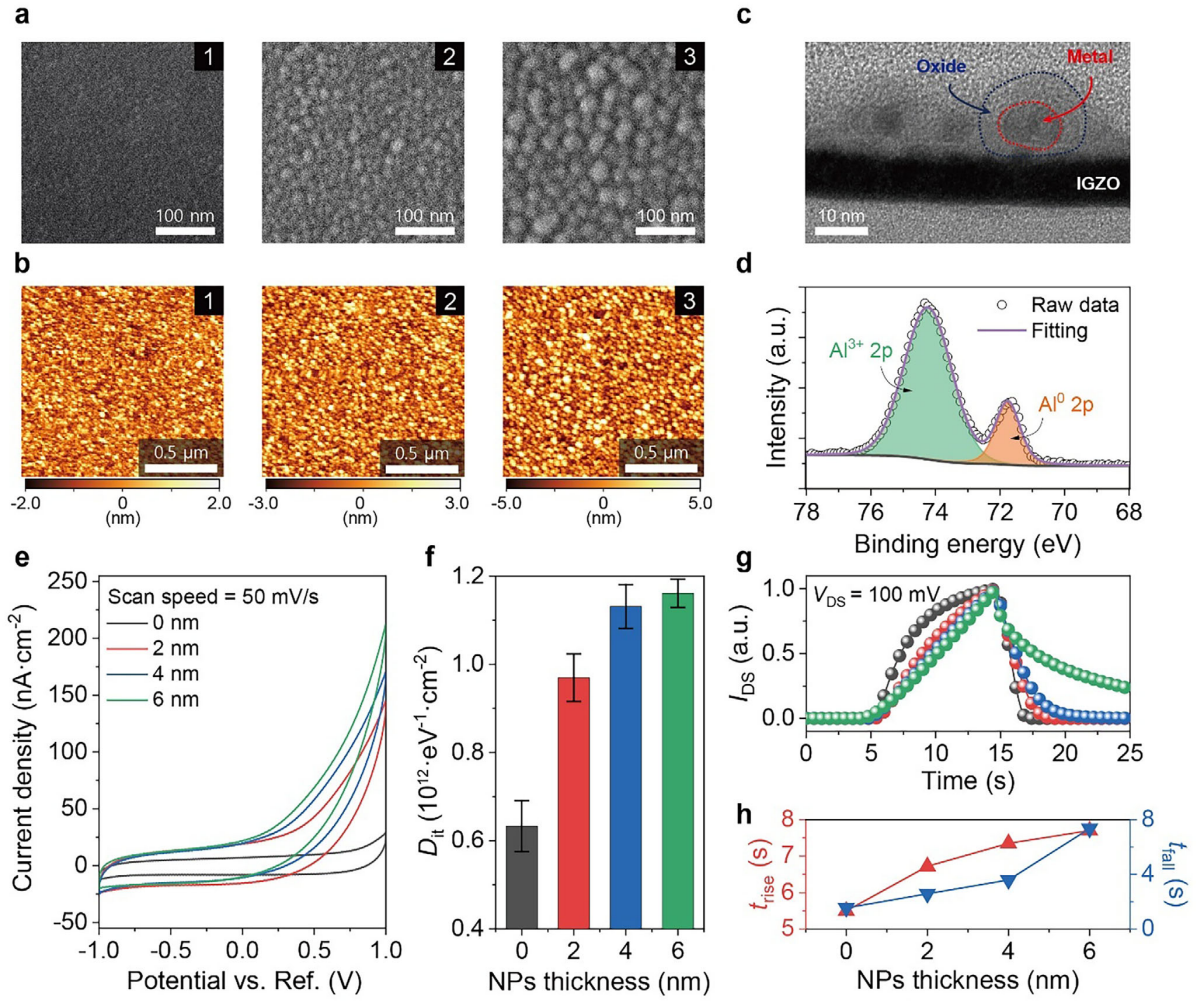
Physicochemical analyses for investigating the thickness effects of Al NPs deposited on the electrolyte–IGZO interface. With different thicknesses of Al NPs (2, 4, and 6 nm), a) scanning electron microscopy images and b) topographic atomic force microscopy images of NP‐decorated IGZO surfaces. c) Cross‐sectional transmission electron microscopy image of the 6‐nm‐thick NP‐decorated IGZO films with discrete boundaries indicating their native oxidation. d) XPS spectra for Al 2*p* deconvoluted into two distinctive stoichiometries, Al^3+^ for native oxide layers and Al° for metallic particles. e) Cyclic voltammetry measurements of NP‐decorated device cells (metal/IGZO–NP/electrolyte/metal) with different NP thicknesses. Each curve was saturated by repetitive scanning over five times. f) Calculated interface trap densities (*D*_it_) and g) normalized transient responses (*I*_DS_) of the EGMTs, with h) rise and fall times, with varying NP thicknesses. The *V*_GS_ was applied at −2 V in the steady state and falling stage, while applied at 2 V in the rising stage. Each response time was defined as the time change between 90% and 10% of *I*_DS_.

A key to the electrical modulation lies in the interface between NP and electrolyte. The surface morphologies of IGZO films with different NP thicknesses were investigated using microscopic analyses. The scanning electron microscopy (SEM) images of NP‐decorated IGZO surfaces demonstrated a lateral growth of the NPs and their discrete boundaries stemming from the native oxidation (**Figure** **2**a). Given the method for NP production,^[^ ^54^, ^55^ ^]^ a thermally evaporated Al layer with a thickness of a few nanometers preferentially forms island‐like grains rather than a continuously densified film because of its high surface energy, thereby functioning as a discrete NPs instead of a conductive layer. Indeed, a continuous conductive pathway emerged at the NP thickness over 8 nm, clearly distinguishing the threshold between the nanoparticulate and metallic behaviors (Figure S5, Supporting Information). The average size and number of particles in the confined area indicate that the size reaches a maximum at 6 nm NPs, while requiring at least 2 nm of thickness to achieve adequate surface coverage and functional NP decoration (Figure S6, Supporting Information). Additionally, the atomic force microscopy (AFM) analysis revealed the omnidirectional growth of NPs and resultant roughness (Figure 2b). Consistent with the SEM results, the grain size increased with the thicker NPs, where the optimal root‐mean‐square (RMS) value increased from 0.71 ± 0.03 nm at 2 nm to 1.16 ± 0.05 nm and 1.68 ± 0.06 nm at 4 nm and 6 nm NPs. The vertical growth of NPs and their native oxidation was further confirmed using transmission electron microscopy (TEM) analysis (Figure 2c). The cross‐sectional TEM image of a 6‐nm‐NP‐decorated IGZO film demonstrated that the particles consist of a crystalline metallic core with diameters on the order of 10 nm and surrounding amorphous shell sufficiently deep for Li^+^ trapping. Despite careful thickness control during evaporation, the actual deposited thickness was found to be slightly greater than the nominal 6 nm, likely due to cumulative effects from surface diffusion and native oxidation during and after deposition.^[^ ^54^ ^]^ The X‐ray photoelectron spectroscopy (XPS) unveiled chemical compositions of the film surface (Figure 2d) The Al 2*p* spectra of the optimally decorated IGZO film was deconvoluted into two distinct peaks: Al^3+^ (binding energy of 74.25 ± 0.05 eV) and metallic Al^0^ (binding energy of 71.40 ± 0.05 eV).^[^ ^56^, ^57^ ^]^ Considering that XPS scans the wide surface of the sample, the distinctive Al^3+^ that corresponds to the Al_2_O_3_ stoichiometry clearly demonstrates native oxidation.

In addition to the understanding of interfacial morphology, electrochemical interpretation effectively supports the mechanistic route for NP‐assisted Li^+^ trapping behaviors. The cyclic voltammetry (CV) measurements on device cells exhibited distinct redox windows associated with Li⁺ trapping (Figure 2e). The integral area that contributes to the total Li^+^ density continued to increase with thicker NPs, attributed to the expanded cavity of the oxide shell in thicker NPs. Meanwhile, the interfacial kinetics were demonstrated by electrochemical impedance spectroscopy (EIS) analysis of parallel and device cells, where the parallel cells included only polymer electrolyte sandwiched by opposite electrodes. Upon the introduction of NP, the interfacial capacitance abruptly increased from 0.755 nF cm^−2^ (0 nm) to 1.13 nF cm^−2^ (2 nm), then gradually decreased to 0.817 nF cm^−2^ (4 nm) and 0.787 nF cm^−2^ (6 nm), respectively (Figure S7 and Tables S2 and S3, Supporting Information). This inversion indicates that the enlarged particle growth with increasing NP thickness reduces the kinetic active sites of Li^+^ at electrolyte interfaces due to limited surface area (Figure S8, Supporting Information). Comprehensively, the enlarged trap volume was dominantly effective in the widening hysteresis window of EGMTs.

The interfacial trap density (*D*_it_) of the EGMTs also proved the thickness‐dependent trap cavity (Figure 2f). The *D*_it_ continuously increased with the thicker NP thicknesses, which is strongly related to the enlarged *S.S*. and expanded *Hys*. values. Furthermore, thickness‐dependent trap effects led to distinctive transient responses of the EGMT. The EGMT with a 6‐nm‐thick NP decoration only exhibited an incomplete recovery after eliminating the positive field (Figure 2g), implying its feasibility for effective learning as memory device. More quantitatively, the extracted response times corroborated that the slower transient responses observed for thicker NPs are likely attributable to the enhanced trapping and delayed release of Li^+^ in the expanded oxide regions (Figure 2h). Consequently, optimized decoration with NPs as high as 6 nm in thickness enlarged the oxide region for effective trap sites, holding promise for expanding synaptic plasticity with extended response times.

### 2.3 Synaptic Functionalities of NP‐Decorated EGMTs with Low Energy Consumption

**Figure 3:**
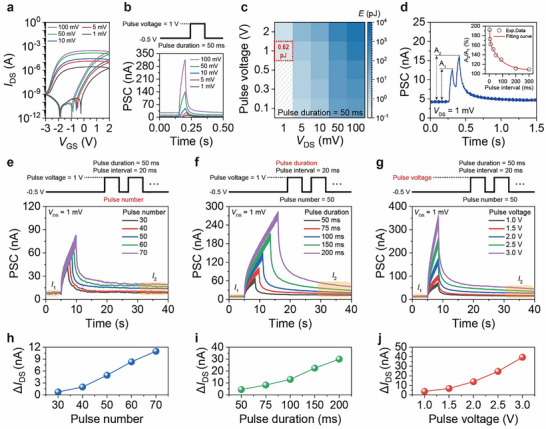
Synaptic behaviors of 6‐nm‐thick NP‐decorated EGMTs with low energy consumption. a) Transfer curves of the EGMT with different *V*_DS_ conditions (100, 50, 10, 5, and 1 mV), and b) the corresponding PSC dynamics stimulated by a single *V*_DS_ at 1 V and *t*_duration_ of 50 ms, where steady‐state voltage was pinned at –0.5 V. c) Energy consumption stimulated by a single pulse, mapped with *V*_pulse_ and *V*_DS_ variations, where the *t*_duration_ was fixed at 50 ms. d) PSC dynamics under 1 mV *V*_DS_ conditions, when the paired pulses were applied with a 50 ms interval. The inset plots the PPF index that is relative amplitude ratio (A_2_/A_1_) triggered by two consecutive spikes, against the altering interval times. PSC dynamics stimulated by consecutive pulses with various e) pulse numbers (30–70 times), f) *t*_duration_ (0–200 ms), and g) *V*_pulse_ (1.0–3.0 V), and Δ*I*_DS_ changes as functions of h) pulse numbers, i) *t*_duration_, and j) *V*_pulse_, where the Δ*I*_DS_ was extracted with the difference between the steady‐state current (*I*_1_) and the current level measured 30 s after the final pulse exertion (*I*_2_).

The brain is composed of a vast network of neurons interconnected by synapses that facilitate information transmission via spike signals.^[^ ^58^ ^]^ For continuous and extensive synaptic interactions, minimizing the energy consumption per spike is essential for low‐power neuromorphic operation. Benchmarked from biological neural networks, achieving low energy consumption in artificial synapses requires minimizing *V*_DS_, because the switching energy follows *E* = *PSC* × *V*_DS_ × *t*_duration_,^[^ ^10^ ^]^ where *PSC* and *t*_duration_ indicate the postsynaptic current and pulse duration, respectively. Owing to Li^+^ trapping and the resultant semi‐reversible responses, all the transfer curves showed a large hysteresis window (**Figure** **3**a). Additionally, a lower *V*_DS_ causes a less effective drift of charge carriers in the IGZO channel, leading to a lower *I*_on_ while maintaining distinct switching characteristics. Comparing the transient response with different *V*_DS_, the PSC levels of the EGMT were monitored by applying a gate pulse voltage (*V*_pulse_) of 1 V with 50 ms of duration (Figure 3b), where the gate bias for steady state was fixed at −0.5 V. Even at extremely low *V*_DS_ down to 1 mV, the EGMT successfully validated its synaptic functionality, albeit with a single spike. By screening various measurement parameters (Figure S9, Supporting Information), such as *V*_DS_, *V*_pulse_, and *t*_duration_, the lowest energy consumption was achieved as low as 0.62 pJ spike^−1^ (Figure 3c). Such a remarkable value was noted by comparing it with those of previously reported MO_x_‐based electrolyte‐gated synaptic devices,^[^ ^10^, ^18^, ^19^, ^20^, ^21^, ^22^, ^23^, ^24^, ^25^, ^26^, ^27^, ^28^, ^29^, ^30^, ^31^, ^32^, ^33^, ^34^, ^35^, ^36^, ^37^, ^38^, ^39^, ^40^, ^41^, ^42^, ^43^, ^44^, ^45^, ^46^ ^]^ which are listed in (Figure S10 and Table S1, Supporting Information).

Under confined pulse conditions with the lowest energy consumption (*V*_DS_ = 1 mV, *V*_pulse_​ = 1 V, *t*_duration​_ = 50 ms), the EGMT demonstrated typical short‐term synaptic plasticity (STSP) featuring paired‐pulse facilitation (PPF) (Figure 3d). These PSC dynamics were triggered by two consecutive gate pulses with pulse interval (*t*_interval_) of 50 ms. To evaluate the validity of the relationship between the PPF index and pulse interval, the experimental data were fitted using a double‐exponential model,^[^ ^26^, ^59^ ^]^ which featured *R* ^2^ > 0.998. The maximum PPF index was estimated to be ≈192% at *t*_interval_ = 2 ms, and it gradually approached 100% as *t*_interval_ increases due to their weakened dependency. These behaviors indicate that the NP‐decorated EGMTs can enable not only long‐term learning but also short‐term transient response, essential for effectively mimicking actual brain functions.

The STSP can be transformed into an LTSP after a series of repeated training sessions in biological systems. Similarly, the application of numerous consecutive gate pulses under various pulse conditions enables the LTSP function of the EGMT. Figure 3e–g shows the dynamics of the PSC by altering the pulse number, duration, and voltage, while the other parameters were fixed. These consecutive gate pulses commonly resulted in the LTSP behavior of the EGMT, which featured a memory‐like function with increased PSC levels (Δ*I*_DS_). So far, the EMGT with a conventional architecture has suffered from achieving such LTSP functions because of the absence of sufficient ion trap sites (Figure S11, Supporting Information), but EGMT decorated with Al NPs successfully demonstrated the LTSP processibility alongside effective STSP function. In addition to the LTSP demonstration, the Δ*I*_DS_ as functions of pulse number, duration, and voltage, remarked their proportional relationship (Figure 3h–j), implying the inherent tradeoff between *DR* and *E*. Whilst maximizing the Δ*I*_DS_ is desired for enlarging *DR* that enables a fine modulation of synaptic weights and enhances accuracies of neuromorphic computation,^[^ ^60^ ^]^ the selection of pulse conditions should have been carefully considered to avoid undesired increase of *E*.

### 2.4 Long‐Term Operation Reliability of Scalable EGMTs

**Figure 4:**
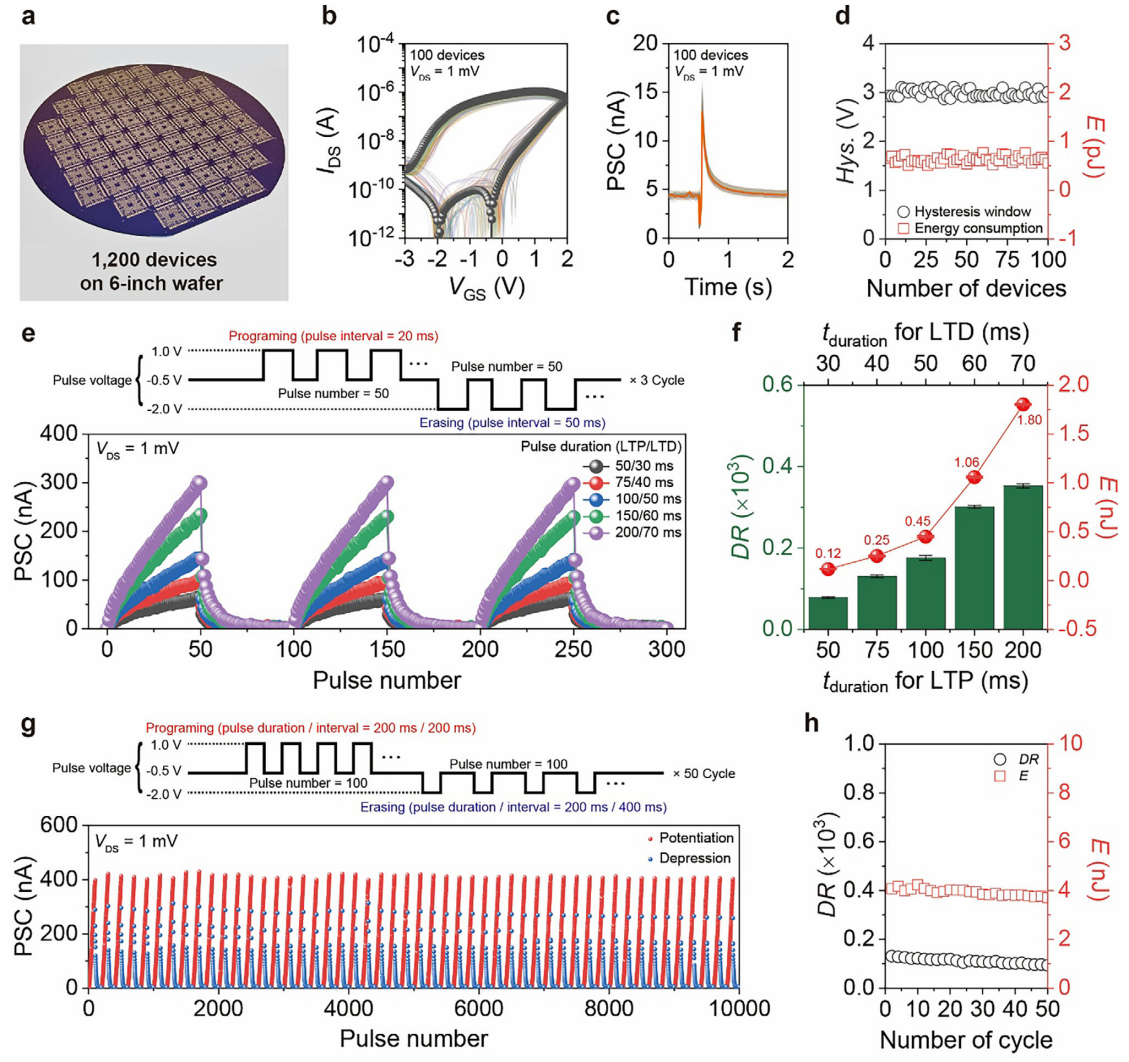
Large‐area fabrication and long‐term operation of the EGMTs. a) Photographic image of EGMTs fabricated on a 6‐inch wafer. b) Transfer curves and c) PSC responses (under single‐pulse stimulation with *t*_duration_ = 50 ms, *V*_pulse_ = 1 V) of 100 randomly selected devices measured at *V*_DS_ = 1 mV; the filled and colored curve in each panel indicates the most representative behavior. d) The extracted *Hys*. and *E* values from 100 devices, demonstrating spatial uniformity and high reliability of the fabrication process. e) LTP–LTD characteristics emulated over three cycles with program/erase pulses under various *t*_duration_ (50–200 ms). f) Plots of *DR* (left) and *E* (right) per single cycle as a function of *t*_duration_. g) PSC dynamics over 10^4^ pulses with a total of 100 LTP–LTD cycles, and h) corresponding *DR* and *E* values extracted as a function of cycle number.

Beyond their low‐energy operation, EGMTs offer a compelling advantage in supporting massive parallel data computation, driven by their scalable fabrication process. To emulate the parallelism of biological neural networks, a high degree of device spatial uniformity is required across large areas. The fabrication processes for EGMT include spin coating of the IGZO film and thermal evaporation of Al NPs, ensuring wafer‐scale producibility (**Figure** **4**a) A total of 60‐unit cells were mounted onto a 6‐inch wafer, where a unit cell contained 20 isolated devices. Among these numerous devices, 100 devices were randomly chosen for electrical characterization to verify their spatial uniformity. The solution‐processed IGZO and thermally evaporated Al NPs successfully ensured not only consistent transfer characteristics across the 6‐inch wafer (Figure 4b), but also the minimized batch‐to‐batch variations (Figure S12). These randomly chosen devices also demonstrated exceptionally low errors in transient pulse response (Figure 4c), and the extracted *Hys*. and *E* values obviously corroborated their statistical uniformity and scalability (Figure 4d). Notably, the uniformity of the hysteresis window plays a crucial role in ensuring the precise modulation of synaptic weights and stable learning characteristics,^[^ ^61^ ^]^ and the consistent energy consumption across devices indicates that the reduced energy consumption can be maintained at scale, supporting reliable low‐power operation in large arrays.

However, for synaptic devices to become reliable alternatives to conventional computing systems, they must exhibit reliable operation over extended periods. This entails consistent synaptic weights without degradation, which can be represented by stable synaptic transitions after repetitive long‐term potentiation (LTP) and depression (LTD) cycles. This is a critical requirement for practical applications requiring continuous learning and memory retention. To verify the long‐term reliability of the EGMT, cumulative excitatory and inhibitory pulses were applied over three cycles under a fixed *V*_DS_ of 1 mV (Figure 4e). Both excitatory and inhibitory pulses represent the programming and erasing steps during the learning stage while modulating the synaptic weight among the EGMTs. Despite the low load of individual pulses, the repetitive PSC dynamics maintained their cyclic shape, indicating their promise for pulse‐reliable learning. The total energy consumption per cycle was plotted as a function of the pulse duration (Figure 4f). The lowest energy consumption for a single cycle was down to 0.12 nJ at *t*_duration_ = 50 ms of LTP. Considering the energy required for processing a single image data (28 pixel × 28 pixel) during learning and inference, which will be discussed in the later section, such sub‐1 nJ energy consumption lies well within the feasible budget for such tasks. With 50 distinguishable conductance (*G*) states achieved within a single LTP–LTD cycle, *DR* values were extracted at 1 mV of *V*_DS_. Because of the intrinsic tradeoff between *E* and *DR*, expressed as *E* = *PSC* × *V*_DS_ × *t*_duration_ and *G* = *PSC*/*V*_DS_,^[^ ^62^ ^]^ achieving fine synaptic weight modulation with low energy consumption is often challenging. Nevertheless, the surface‐engineered IGZO EGMTs enabled facile Li^+^ trapping with a significantly enlarged *Hys*., which effectively enhanced *G*_max_ and suppressed *G*_min_, yielding a notable *DR* as high as 78 even at 1 mV of *V*_DS_ while maintaining a low *E* of 0.12 nJ.

The operational reliability of the EGMT was evaluated under various practical conditions. The consecutive transitions over 10 000 pulses of potentiation (*V*_GS_ = 1 V) and depression (*V*_GS_ = −2 V) were applied to the device with a total of 50 cycles of input pulses (Figure 4g), which ensured the consistent cyclic performance, while the extracted *DR* and *E* also exhibited no significant degradation over 50 cycles of pulse training (Figure 4h). In addition to the cyclic reliability, the EGMT demonstrated outstanding environmental resilience under varying temperature and relative humidity (Figure S13 and Table S4, Supporting Information). This environmental robustness can be attributed to the long‐term stable polymer electrolyte of which ionic conductivity and device performance maintained in ambient air up to 10 days (Figures S14 and S15, Supporting Information). Consequently, the NP‐decorated EGMT achieved long‐term operational stability under practical conditions, coupled with its environmental robustness, proposing the seamless integration of neuromorphic devices into daily life.

### 2.5 Image Data Learning and Inference with Artificial Neural Network Simulations

**Figure 5:**
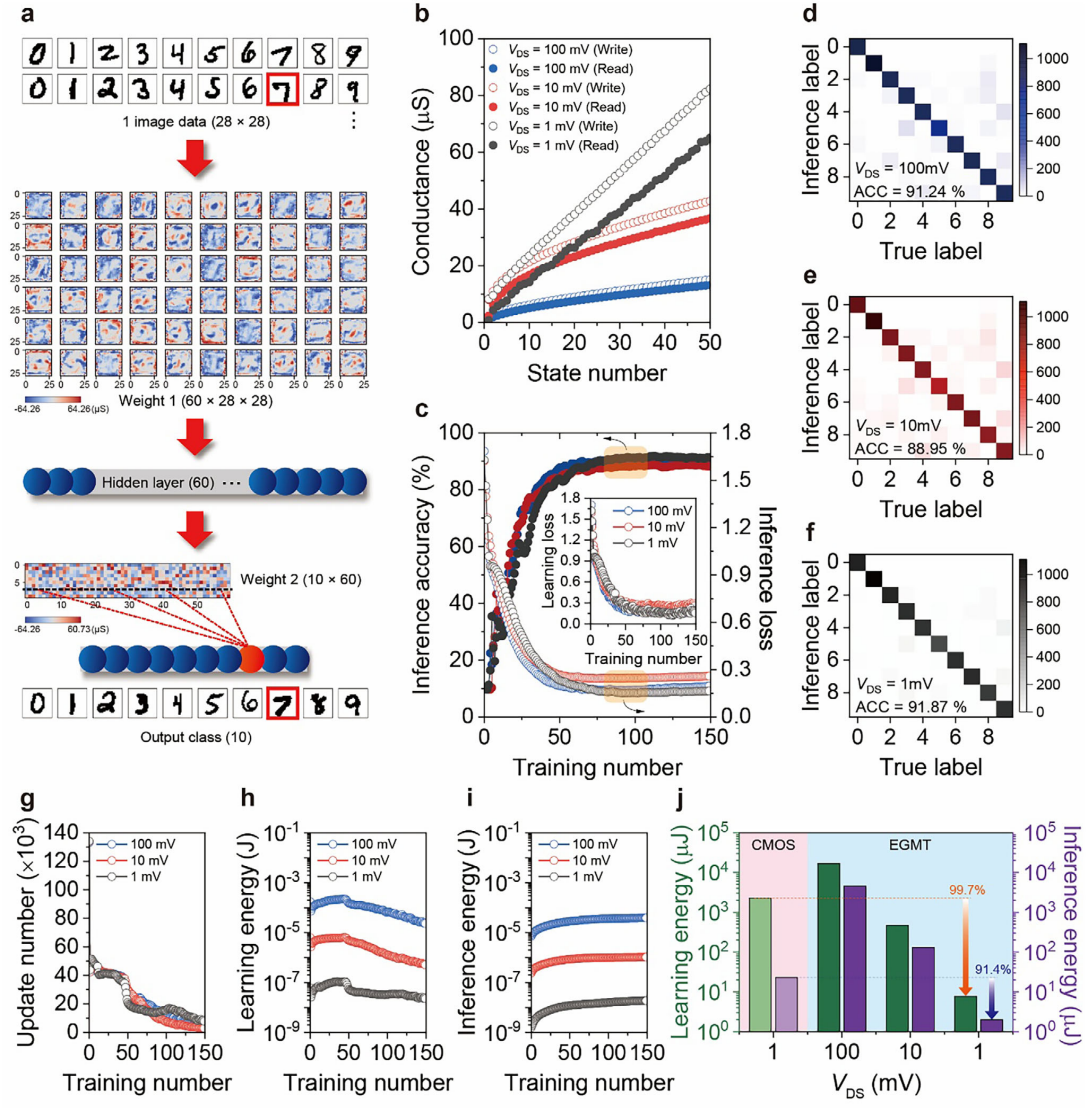
Neuromorphic analysis. a) The 784 × 60 × 10 multi‐layer neural network architecture where 28 × 28 MNIST image can be transformed through 60 × 28 × 28‐dimensional first weight and 10 × 60‐dimensional second weight to determine the output class among 10‐digit labels and to evaluate the loss value. b) The analog conductance value defined as PSC divided by *V*_DS_ at the write scheme (empty circle) and read scheme (filled circle) according to potentiation state number for the different *V*_DS_ values of 100, 10, and 1 mV. c) Inference accuracy (left axis) and inference loss value (right axis) for 10 000 inference MNIST images during the training process for the different *V*_DS_ values of 100, 10, and 1 mV. The inset figure shows the learning loss value for 500 learning MNIST images, which are different for each training epoch. Confusion matrices for 10 000 inference images for the different *V*_DS_ values of d) 100 mV, e) 10 mV, and f) 1 mV, where the row indicates the true label and column indicates inferred label among ten output classes. Energy consumption analysis as g) weight update number, h) learning energy, and i) inference energy for one 28 × 28 image processing during the training process for the different *V*_DS_ values of 100, 10, and 1 mV. j) Total energy consumption value after the 150 training epochs for learning energy (left green bar) and inference energy (right purple bar) for different weight programming schemes such as CMOS, *V*_DS_ values of 100, 10, and 1 mV.

**Figure** **5**a shows the neural network architecture utilized to evaluate the nonvolatile and multistate conductance modulation properties of EGMTs to perceptron tasks for unstructured data processing. The corresponding weight map was extracted at 1 mV of *V*_DS_ to minimize energy consumption. Additional weight maps were obtained at elevated *V*_DS_ values of 10 and 100 mV (Figure S16, Supporting Information). All synaptic weight elements in the 784 × 60 × 10 multilayer neural networks were designed with discrete actual analog conductance values among the 50 multistate levels of EGMTs. The binarized 28 × 28 modified National Institute of Standards and Technology (MNIST) dataset was transformed through a 60 × 28 × 28 first weight into 60 hidden layers, followed by transformation through a 10 × 60 s weight to determine the designated output class among ten labels using the maximum activation value and to evaluate the learning loss value. The detailed procedures for learning and inference are discussed in the Experimental Section. During training, synaptic weights were iteratively updated via batch‐mode backpropagation to minimize loss and enhance inference accuracy. Because the hardware‐level analog synaptic weight depends entirely on the multilevel trajectory of the conductance states according to the constant voltage potentiation process of EGMTs, it is important to satisfy the uniform LTP process, excluding programming or read noise.

Figure 5b shows the distinct conductance trajectories for different *V*_DS_ values. Notably, the read and write modes indicate the states for −0.5 and 1.0 V of *V*_GS_ applied, respectively, during the LTP processes. When *V*_DS_ was maintained at 1 mV during gate potentiation, the read and write conductance values exhibited more linear trajectories with a nonlinearity of 0.53, in contrast to the higher values exceeding 1 observed at *V*_DS_ values of 10 and 100 mV. Because the conductance value was inversely proportional to the read voltage, the device potentiated at 1 mV *V*_DS_ showed the highest conductance despite having the lowest PSC value compared to the devices potentiated at 10 and 100 mV (Figure S17, Supporting Information). Furthermore, the conductance values in the write mode were temporarily higher than those in the read mode for all *V*_DS_ cases, indicating that programming requires more energy than reading in multistate conductance‐switching memory operations. This difference may be partially attributed to the longer pulse duration during programming.^[^ ^12^, ^63^ ^]^ Because the potentiation curves at 1 mV *V*_DS_ showed a near‐ideal multi‐state trajectory under a constant programming voltage with the highest linearity and large *DR* during LTP, the perceptron model also demonstrated superior performance at 1 mV of *V*_DS_, showing higher accuracy and lower inference loss compared to the 10 and 100 mV *V*_DS_ conditions (Figure 5c). Despite nonlinear LTP–LTD switching, the conductance‐based emulation for the synaptic weight was well implemented according to the backpropagation learning rule because the unit weight consists of a pair‐cell (positive and negative) structure.^[^ ^64^ ^]^ Here, both increases and decreases in the synaptic weight can be actualized using only the LTP process, with high linearity during the training process. Specifically, the positive cell is potentiated if the weight is increased, and the negative cell is potentiated if the weight is decreased for specific synaptic weight components (see the Experimental Section).

Consequently, as shown in Figure 5d–f, the inference accuracy for the 10 000‐sample test dataset was ≈91.87 % at 1 mV of *V*_DS_ condition, which was considerably higher than the accuracies achieved at 10 mV (≈88.95 %) and 100 mV (≈91.24 %). In addition, the learning and inference loss values at 1 mV of *V*_DS_ showed ≈0.118 and 0.152, respectively, which were superior to those at 10 mV (≈0.217 and ≈0.244) and 100 mV (≈0.143 and ≈0.169). In addition to binary MNIST, we extended the evaluation to analog MNIST and analog Fashion‐MNIST (F‐MNIST) datasets to demonstrate evaluation across different image types and data representations (Figure S18, Supporting Information). Both analog MNIST and analog F‐MNIST show the grayscale image with 256‐level complexity. Because analog images possess the enhanced precision compared to binary images (0 or 1), the inference accuracy also showed an improved value of ≈92.04 % (Figure S19a, Supporting Information) compared to that in binary MNIST. Additionally, loss value during learning stage and inference tasks showed ≈0.115 and ≈0.141 in analog MNIST, providing improved value compared to the results in binary MNIST. In case of analog F‐MNIST, the complexity of the dataset showed inference accuracy as ≈81.24 % (Figure S19b, Supporting Information) and loss value for learning and inference as ≈0.192 and ≈0.202, respectively. Consistently, additional weight maps obtained for analog MNIST and analog F‐MNIST datasets (Figure S20, Supporting Information). Figure 5g shows the updated number of the intermediate conductance states of the EGMTs for different *V*_DS_ conditions at 100, 10, and 1 mV. For all programming conditions, the number of weight updates decreased as training progressed owing to the nature of backpropagation, which gradually led to saturation in the weight configurations. That is, once the synaptic weights in the neural network are sufficiently trained, further updates become infrequent in the later stages.^[^ ^65^ ^]^

Despite the similar number of weight updates across different *V*_DS_ conditions, the total energy consumption during training varied with *V*_DS_ conditions because the energy required for each update depended on the conductance level and pulse duration. Their dependency during learning is plotted in Figure 5h, which was calculated by the *V*_DS_^2^ × *G* (temporal transition level during LTP) × *t*_duration_, resulting in higher energy consumption at higher *V*_DS_ values. After 150 training cycles, the total learning energy was analyzed by summing the energy consumed at each training step, yielding 7.67 µJ under 1 mV, 4.71 × 10^2^ µJ under 10 mV, and 1.66 × 10^4^ µJ under 100 mV *V*_DS_ conditions, respectively. During the early training cycles, the learning energy increased owing to a high number of weight updates accompanied by increasing conductance levels; however, the energy consumed per training step began to decrease after 50 training epochs, primarily owing to an abrupt reduction in the number of weight updates, despite a continued overall increase in conductance. In contrast, the inference energy increased monotonically owing to the increasing conductance levels, as shown in Figure 5i. This is because the total number of vector‐matrix multiplication operations was fixed using the 10 000‐sample inference dataset. The read voltage and pulse duration (20 ms) were kept constant throughout the training steps, whereas the overall conductance values in the weight map gradually increased. The overall inference energy showed 1.98 µJ under 1 mV, 1.31 × 10^2^ µJ under 10 mV, and 4.57 × 10^3^ µJ under 100 mV of *V*_DS_ conditions, respectively. The *V*_DS_‐dependent total learning and inference energy consumption is summarized in Figure 5j. Here, the energy consumption in CMOS digital operation was also obtained in identically fixed 784 × 60 × 10 network architecture with floating number weight operation, where the learning and inference energy showed 2.28 × 10^3^ and 2.29 × 10^1^ µJ, respectively.^[^ ^66^ ^]^ More explicitly, when energy efficiency was evaluated in terms of the number of operations performed over 150 training epochs per unit inference energy, the EGMT under 1 mV *V*_DS_ condition achieved 3.61 tera‐operations per second per watt (TOPS)/W, whereas the 32‐bit CMOS implementation yielded 0.31 TOPS/W. Note that the energy efficiency can be obtained simply by dividing the total number of operations by its inference energy consumption. This substantial difference indicates that the EGMT provides roughly an order‐of‐magnitude higher inference efficiency than the digital CMOS baseline, further reinforcing its advantage for energy‐efficient neuromorphic processing. The reduced energy consumption was also observed in analog MNIST and analog F‐MNIST datasets at *V*_DS_ = 1mV (Table S5, Supporting Information). For example, learning energy values were calculated as 7.85 µJ for analog MNIST and 8.39 µJ for analog F‐MNIST, which is similar to that in binary MNIST (7.67 µJ). Also, inference energy values were obtained as 1.37 µJ for analog MNIST and 2.31 µJ for analog F‐MNIST, which is also similar to that in binary MNIST (1.98 µJ). Because energy consumption in CMOS operation is seldom affected by the dataset when the image size is fixed, the decreasing rate of the energy showed similar results as in binary MNIST in spite of differences between dataset variability. Compared with the energy consumption of 32‐bit CMOS implementation (Tables S6 and S7, Supporting Information), EGMTs demonstrated ≈99.7% and ≈91.4% reductions in training and inference energy, respectively, highlighting their significant potential for energy‐efficient neuromorphic computing.

## 3 Conclusion

This study developed scalable EGMTs incorporating Al NP as ion trap sites to lower energy consumption and expand *DR*. By integrating a polymer electrolyte (PEO:LiAsF_6_) with a solution‐processed IGZO channel, 1 mV of *V*_DS_ enabled record‐level low energy consumption of EGMTs, down to 0.62 pJ spike^−1^. The devices successfully demonstrated synaptic behaviors involving both STSP and LTSP. The robust polymer electrolyte guaranteed electrochemical and environmental stability over extended periods, and thereby, the devices ensured long‐term and repetitive operational reliability as well as high spatial uniformity across a 6‐inch wafer, proving their feasible identity as neuromorphic hardware in practical applications. Neural network simulations using a multilayer perceptron architecture confirmed that reduced energy consumption translated into system‐level savings, showing a 99.7% reduction in training energy and 91.4% reduction in inference energy compared to conventional CMOS implementations. These results indicate that the proposed EGMT platform is a scalable and energy‐efficient solution for neuromorphic computing, with promising implications for next‐generation AI hardware, brain‐inspired systems, and edge intelligence. Future research will focus on material‐level optimization and system integration to further advance scalability and multifunctionality.

## 4 Experimental Section

### Materials

Indium nitrate hydrate (In(NO_3_)_3_ ∙ xH_2_O), gallium nitrate hydrate (Ga(NO_3_)_3_ ∙ xH_2_O), zinc acetate dehydrate (Zn(CH_3_COO)_2_ ∙ 2H_2_O) and 2‐methoxyethanol were purchased from Sigma–Aldrich (St. Louis, MO, USA) and used without further purification. PEO ((‐CH_2_CH_2_O‐)_n_) and Lithium hexafluoroarsenate (V) (LiAsF_6_) were purchased from Thermo Fisher Scientific (Waltham, MA, USA). Acetonitrile (CH_3_CN) was also purchased from Sigma–Aldrich (St. Louis, MO, USA).

### Materials Preparation

To prepare the IGZO solutions, each precursor was dissolved in 2‐methoxyethanol at the following composition ratio (In:Ga:Zn = 7:1:2), while the concentration of the total metal ions was fixed at 0.1 m. The IGZO solution was vigorously stirred for 4 h at 60 °C. PEO and LiAsF_6_ were dissolved in acetonitrile to prepare a polymer electrolyte solution with a concentration of 3 wt.% and mixed in the following mass ratio (PEO:LiAsF_6_ = 9:1). The mixture was then stirred at room‐temperature (≈25 °C) for 24 h.

### Fabrication of EGMTs with Al NPs

A thermally oxidized SiO_2_ layer with a thickness of 300 nm was prepared using heavily boron‐doped Si wafers as substrates. Titanium (Ti/10 nm) and gold (Au/50 nm) were sequentially deposited using an e‐beam evaporator and patterned using conventional photolithography to form the bottom contacts. Prior to spin‐coating the IGZO solution, an oxygen plasma treatment was conducted at 100 W for 10 min (Femto Science, Korea) to obtain a hydrophilic surface. The IGZO solution was spin‐coated at 4000 rpm for 30 s. The samples were annealed at 400 °C for 1.5 h for densification and condensation, followed by patterned using photolithography and a wet etching process. After the deposition of SU‐8 3008 as the passivation layer, Al NPs were deposited on the IGZO channel via thermal evaporation. Subsequently, 100 µL of polymer electrolyte solution was dropped onto the active site. The final sample was then dried under vacuum for 24 h at RT.

### Electrical Characterizations

Electrical characterization of the transfer curves was conducted under ambient conditions using a Keithley 2602 B dual‐channel source meter (Keithley, Cleveland, USA). The synaptic properties of the EGMTs were measured under ambient conditions using a Keithley 4200A source‐meter system with customized transistor characterization software. Postsynaptic current behavior as a function of variable pulses was monitored using a PMU pulse generator connected to the source meter.

### Neural Network Simulation with MLP

As an unstructured dataset to evaluate the neuromorphic application of synaptic devices, 28 × 28 MNIST images were prepared as totally separated 60 000 learning images (5923, 6742, 5958, 6131, 5842, 5421, 5918, 6265, 5851, 5949 for each digit class) and 10 000 inference images (980, 1135, 1032, 1010, 982, 892, 958, 1028, 974, 1009 for each digit class) at the binarized level (0 or 1).^[^ ^67^ ^]^ For one training epoch, 500 MNIST images (50 images per digit label, differently selected for each epoch) were individually transformed through weight 1 (60 × 28 × 28), followed by sigmoid transformation to 60 hidden layer elements, transformation through weight 2 (10 × 60), and a second sigmoid transformation (Figure 5a) to determine the output class among 10‐digit labels in supervised learning by the maximum output decision principle and to evaluate the loss value by mean‐squared error calculation. The synaptic weight for weight 1 (60 × 28 × 28) and weight 2 (10 × 60) which consist of pair‐cell structure (*w* = *G* ^+^ – *G* ^–^) to reflect both positive and negative weight value by using positive conductance level at 50‐state number (Figure 5b)^[^ ^64^ ^]^ was updated by conventional backpropagation principle in batch‐mode^[^ ^65^ ^]^ according to sign and magnitude of partial derivative from weight (*w*) to loss value (*z*) as ∂*z/*∂*w*. Concretely, if ∂*z/*∂*w* is negative value, the positive cell should be updated to increase the weight value, and if ∂*z/*∂*w* is positive value, the negative cell should be updated to decrease the weight value until the conductance value reach the maximum 50th state, where the purpose of weight adjustment is laying on the reducing the learning loss value; this would finally lead the reduction of inference loss and increase of inference accuracy (see Figure 5c). Furthermore, the number of weight updates was uniformly distributed according to the relative magnitude of ∂*z/*∂*w* value from zero to the maximum ∂*z/*∂*w* value to provide three cases of potentiation number (+ 1, + 2, and + 3) for each synaptic weight element at one training epoch as a delta rule application in the stochastic gradient descent of the backpropagation update rule in terms of the hardware analog weight level.^[^ ^68^ ^]^

### Energy Consumption Analysis for Training Process

To evaluate the learning energy during the training process, it was necessary to obtain the weight update number using the batch‐mode back‐propagation update rule based on ∂*z/*∂*w* value. Because the hardware weight was simulated in both the positive and negative cells for weight 1 (60 × 28 × 28) and weight 2 (10 × 60), the total number of weight elements was 2 × (60 × 28 × 28 + 10 × 60), which was ≈9.5 × 10^4^. In addition, the number of weight updates can be determined in three cases (+ 1, + 2, and + 3) according to the magnitude of ∂*z/*∂*w* value; that is, the available maximum weight update per training epoch is approximately 3 × 9.5 × 10^4^ (≈2.85 × 10^5^). Of course, because not all weights would participate in potentiation, the initial number of weight updates was ≈5 × 10^4^ per training epoch, which gradually decreased as the weight map was saturated in the backpropagation update (see Figure 5g). The learning energy in the programming scheme (*V*_GS_ = 1 V) can be obtained from the product of *V*_DS_^2^ (*V*_DS_ = 1, 10, and 100 mV), *G* value during the potentiation process (temporal transition level, empty circle in Figure 5b), and the writing pulse time (50 ms), which are summed for all weight elements (≈9.5 × 10^4^) for each training epoch. Similarly, the inference energy could be obtained in the same *V*_DS_ range (1, 10, and 100 mV) as the *G* value during the read process (stable analog level, filled circle in Figure 5b) and read pulse time (20 ms), which were averaged for 10 000 inference images for each training epoch to evaluate the energy consumption value at one 28 × 28 image data processing. For fair comparison with conventional CMOS computing, the reference CMOS parameters were adopted from previous study,^[^ ^66^ ^]^ which provides standard energy‐per‐operation data widely used in neuromorphic benchmarking. The CMOS baseline was defined as a 45 nm technology node operating at 0.9 V supply with 32‐bit floating‐point precision. The energy cost per 32‐bit memory access was taken as 20 pJ, and the energy per 32‐bit vector–matrix multiplication (VMM) was set to 3.2 pJ. Both systems were assumed to execute equivalent VMM workloads under identical network architectures and task sizes (60 × 28 × 28 + 10 × 60 operations). The CMOS baseline employed a 32‐bit digital arithmetic model, while the EGMT‐based simulation utilized analog conductance updates directly extracted from experimental measurements. This framework enables a direct, operation‐level comparison of intrinsic energy efficiency between digital and analog computing elements, independent of technology node or circuit complexity.

## Conflict of Interest

The authors declare no conflict of interest.

## Supporting information



Supporting Information

## Data Availability

The data that support the findings of this study are available from the corresponding author upon reasonable request.
