# Supplementary material for: Al Nanoparticle‐Decorated Metal Oxide Synaptic Transistors for Ultralow‐Energy Neuromorphic Computing with Wide Dynamic Range
Source: Adv Sci (Weinh). 2025 Nov 30;13(9):e21321. doi: 10.1002/advs.202521321 (PMC12904040; doi:10.1002/advs.202521321)
Supplement: Supplementary file 1 — Supporting Information [file ADVS-13-e21321-s001.docx]

Supporting Information of

**Al Nanoparticle-Decorated Metal Oxide Synaptic Transistors for Ultralow-Energy Neuromorphic Computing with Wide Dynamic Range**

*Jun-Gyu Choi*^1†^*, Yoonseok Song*^2†^*, Seokhyeon Baek*^2†^*, Jingon Jang*^3^**, and Sungjun Park*^1,2^****

^1^Department of Electrical and Computer Engineering, Ajou University, Suwon 16499, Republic of Korea

^2^Department of Intelligence Semiconductor Engineering, Ajou University, Suwon 16499, Republic of Korea
^3^School of Computer and Information Engineering, Kwangwoon University, Seoul 01897, Republic of Korea

^†^These authors contributed equally.

*Correspondence: jingonjang@kw.ac.kr

**Correspondence: sj0223park@ajou.ac.kr

**Keywords:** neuromorphic device, low-energy consumption, synaptic plasticity

**
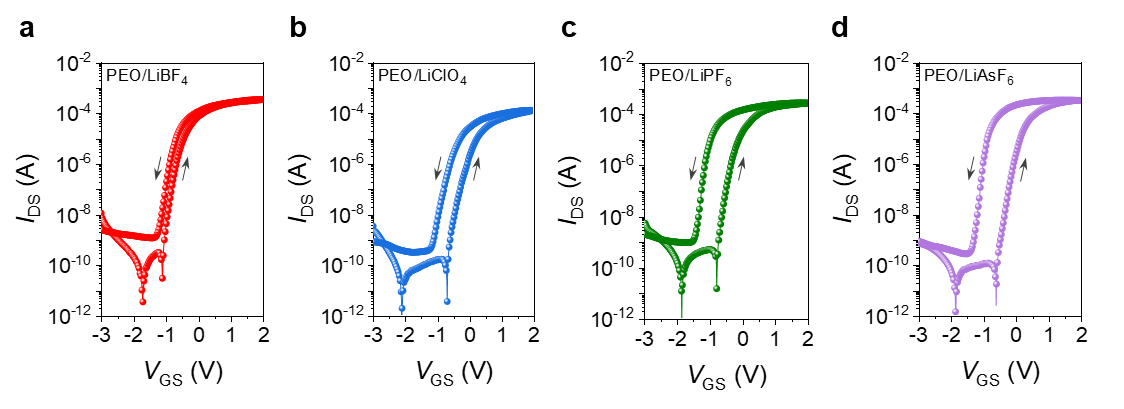
**

**Figure S1**. Measurement of transfer curves depending on anionic species. Representative transfer curves, in which electrolyte is based on PEO with (a) LiBF_4_, (b) LiClO_4_, (c) LiPF_6_, and (d) LiAsF_6_ as a function of the gate voltage (*V*_GS_: -3 to 2 V) and drain voltage (*V*_DS_: 100 mV). Anticlockwise hysteresis windows are extracted with the *V*_GS_ difference maximum value, indicating different ionic mobility of electrolyte. The order of ionic mobility is LiAsF_6_ < LiPF_6_ < LiClO_4_ < LiBF_4_. LiBF_4_ is faster than LiAsF_6_ in formation of EDL, giving a rise to lower hysteresis in the *I*-*V* curve.


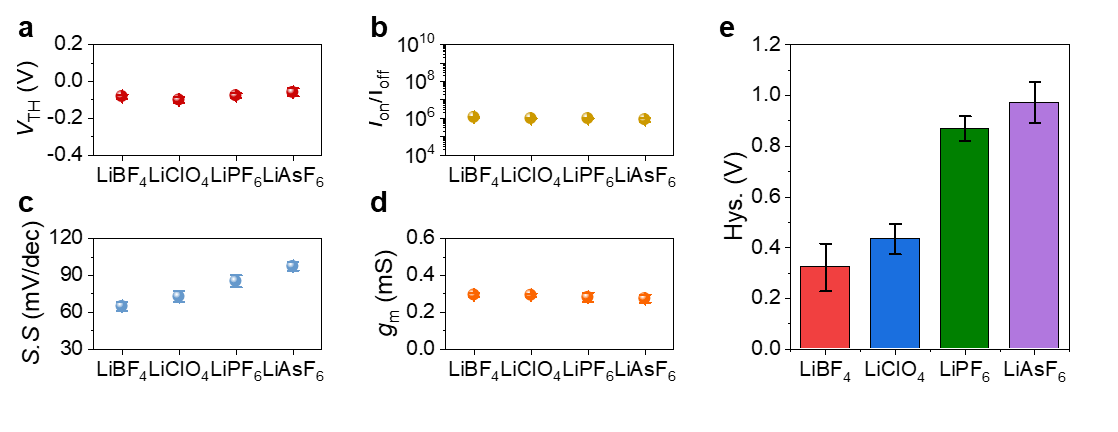


**Figure S2**. Semiconductor parameter extraction of (a) threshold voltage, (b) on/off current ratio, (c) subthreshold swing, and (d) transconductance; error bars denote standard deviations over a total of 10 devices. (e) Average values of each hysteresis window out of 10 devices.


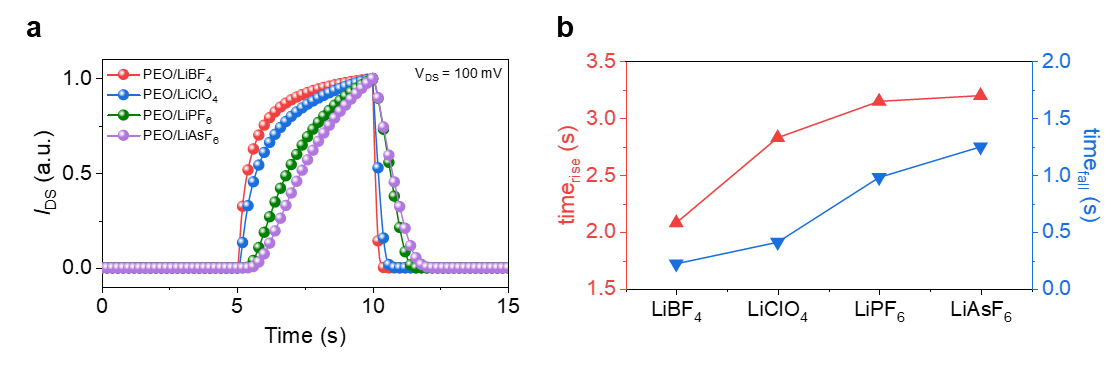


**Figure S3**. (a) Normalized transient response of the synaptic transistors with different anions. *V*_GS_ was applied at -1 V in steady state and falling stage, while 1 V of *V*_GS_ was applied in rising stage. (b) Rise (left) and fall (right) time of each material. Each response time was defined as the time change between 90% and 10% of *I*_DS_.


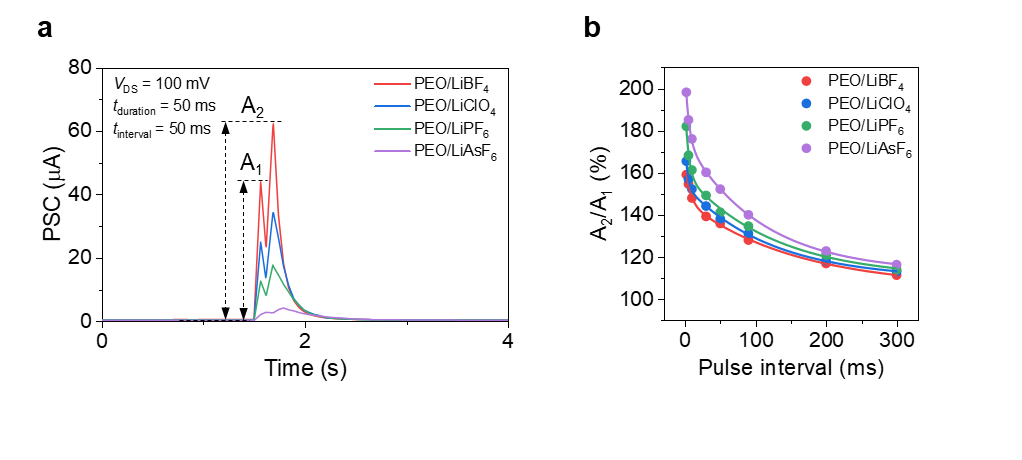


**Figure S4**. (a) Paired-pulse facilitation (PPF) characteristics of each material (*t*_duration_ = 50 ms, *t*_interval_ = 50ms). (b) PPF index, defined as the ratio of A_2_/A_1_, is plotted as a function of pulse interval between two consecutive pre-synaptic spikes.

**
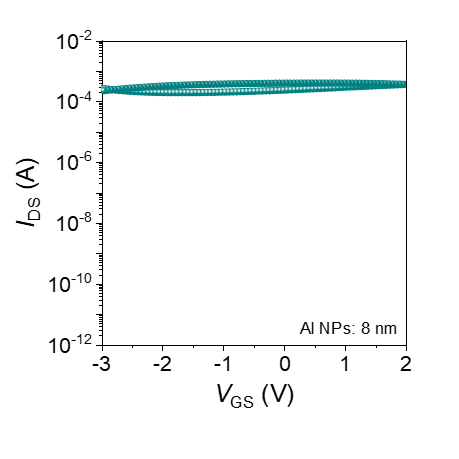
**

**Figure S5**. Transfer curve, in which electrolyte is based on PEO:LiAsF_6_ with 8 nm Al NPs as a function of the gate voltage (*V*_GS_: -3 to 2 V) and drain voltage (*V*_DS_: 100 mV).


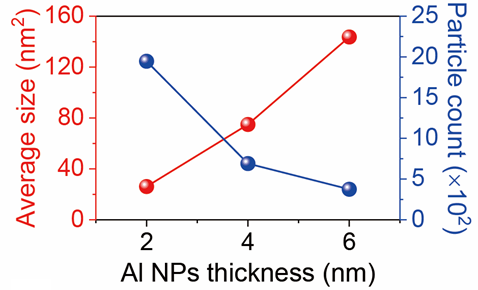


**Figure S6**. Average sizes (left) and count number (right) of particles on the IGZO surfaces, where those in SEM images were counted by Image J software.


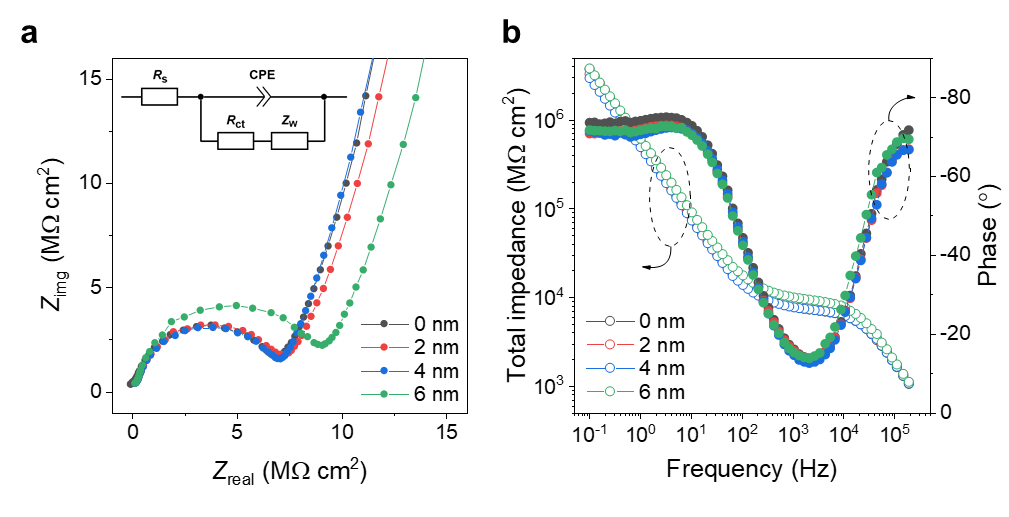


**Figure S7**. Impedance analysis of NP-engineered device cells by electrochemical impedance spectroscopy (EIS) measurement. (a) Nyquist plots with the corresponding equivalent circuit diagram demonstrate individual impedance parameters and (b) Bode plots support the kinetics of the cells, where *R*_s_, *R*_ct_, *Z*_W_, and *CPE* indicate solution resistance through the electrolyte, charge transfer resistance, Warburg impedance, and constant phase element. Given the gate voltage window of device cell for operation, the DC bias was fixed at 0 V, where AC voltage was 10 mV.

**
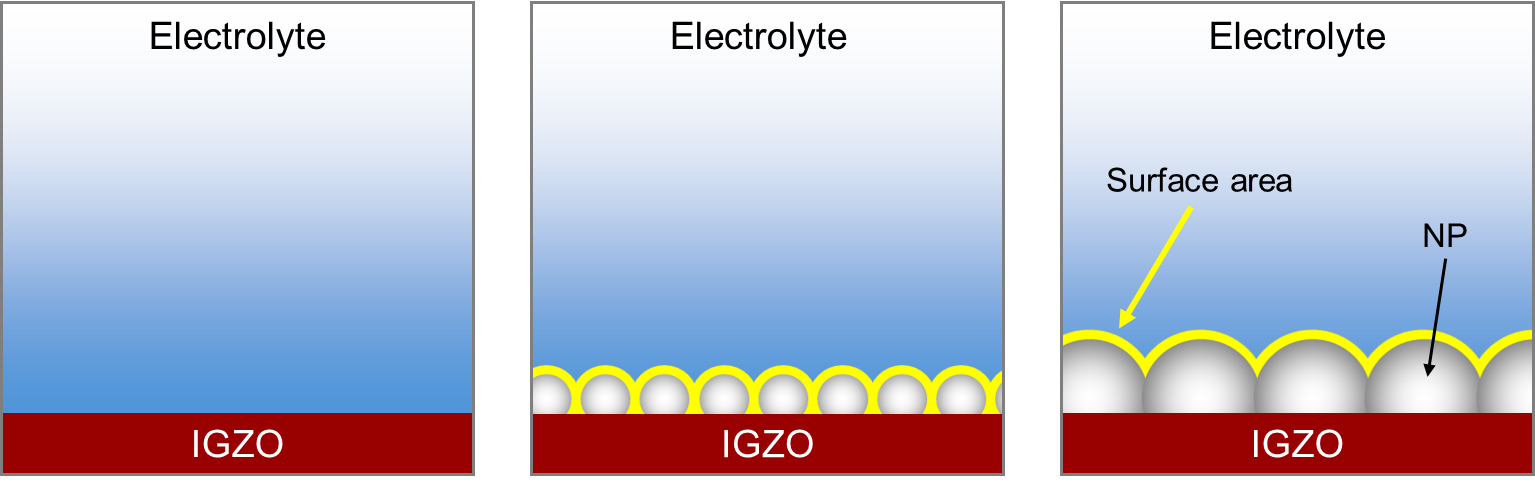
**

**Figure S8**. Schematic of the interface between electrolyte and NP-engineered IGZO with different NP thicknesses (left, 0 nm; center, 2–4 nm; right, 6 nm). The particle growth reduces the surface area of NPs that serve as kinetically active reaction sites.


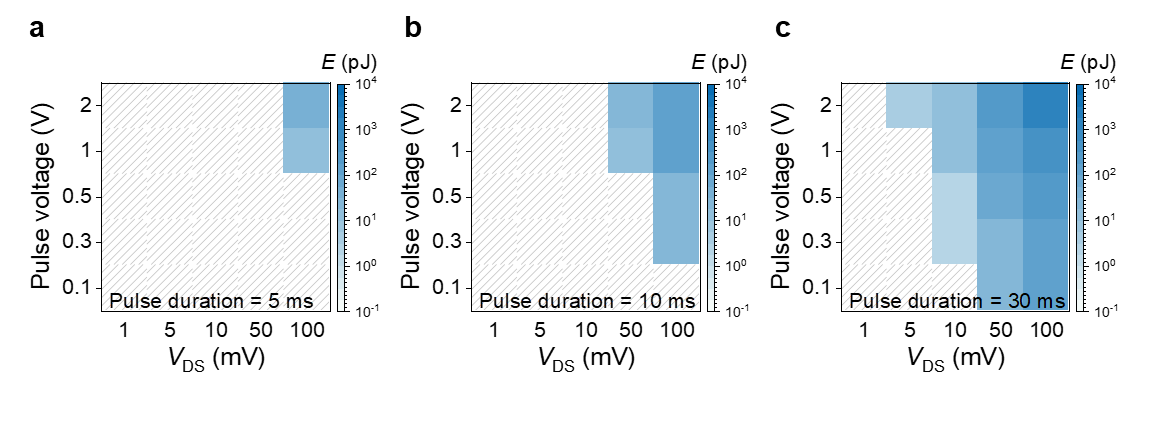


**Figure S9**. (a) Energy consumption under the following conditions: pulse voltage, drain voltage variation, and fixed pulse duration = (a) 5 ms, (b) 10 ms, and (c) 30 ms.

**
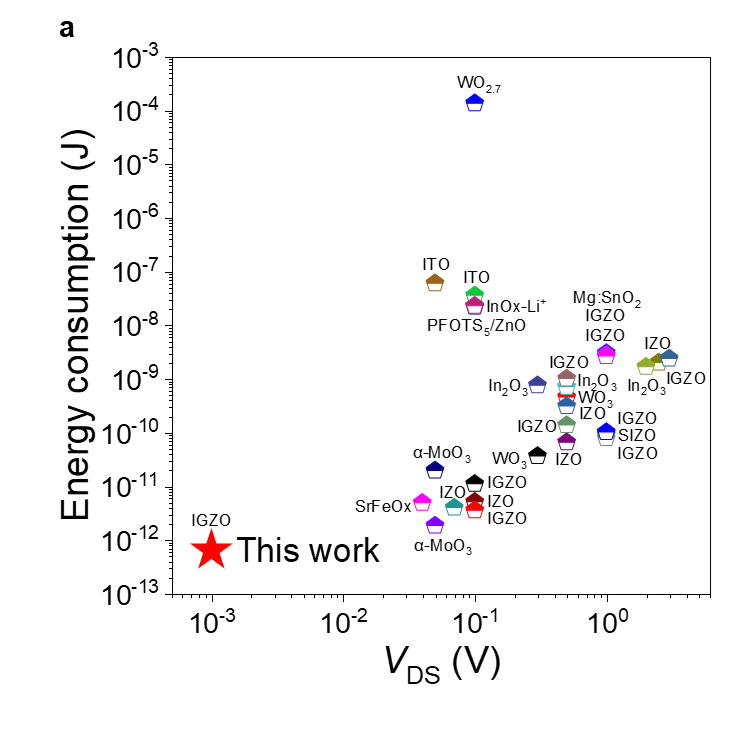

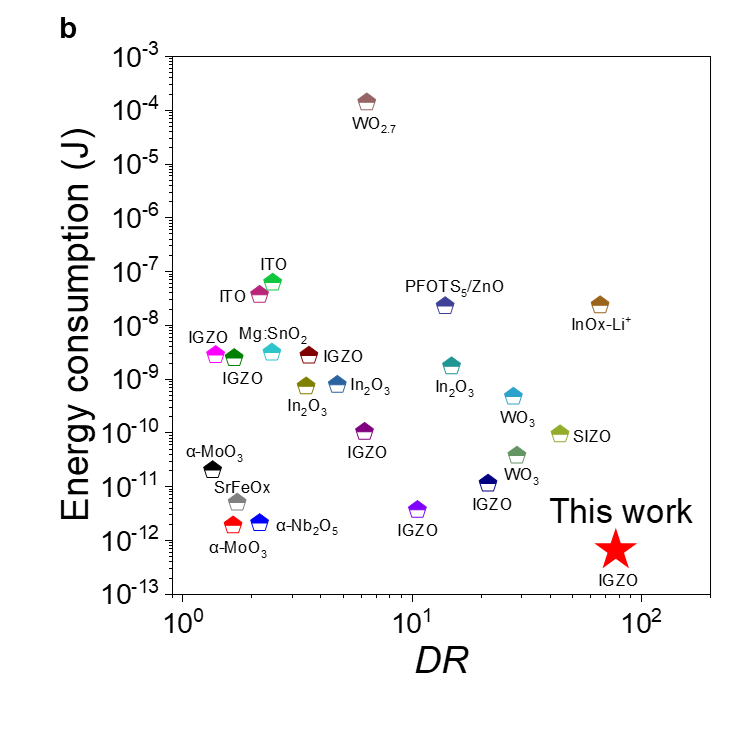
**

**Figure S10**. Figure-of-merit (energy consumption per spike depending on *V*_DS_ and *DR*) for state-of-art neuromorphic devices in the recently reported literature, among planar-structured metal-oxide channel-based electrolyte-gated thin-film transistors. (α-MoO_3_: alpha-molybdenum trioxide, α-Nb_2_O_5_: alpha-niobium pentoxide, IGZO: indium gallium zinc oxide, In_2_O_3_: indium (III) oxide, InOx-Li^+^: lithium-doped indium oxide, ITO: indium tin oxide, IZO: indium zinc oxide, Mg:SnO_2_: magnesium-doped tin (IV) oxide, PFOTS_5_/ZnO: 1H,1H,2H,2H-perfluorooctyltriethoxysilane-treated zinc oxide, SIZO: silicon indium zinc oxide, SrFeOx: strontium iron oxide, WO_2.7_: tungsten suboxide, WO_3_: tungsten trioxide)


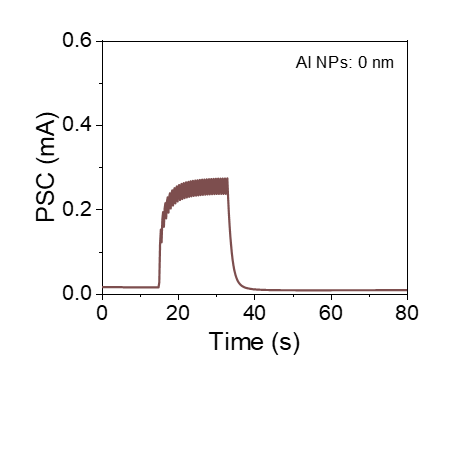


**Figure S11**. PSC response of the EGMT without Al NP-decoration, stimulated by 30 consecutive gate pulses (pulse duration = 400 ms, pulse interval = 200 ms, pulse voltage = 2 V) under *V*_DS_ = 100 mV.


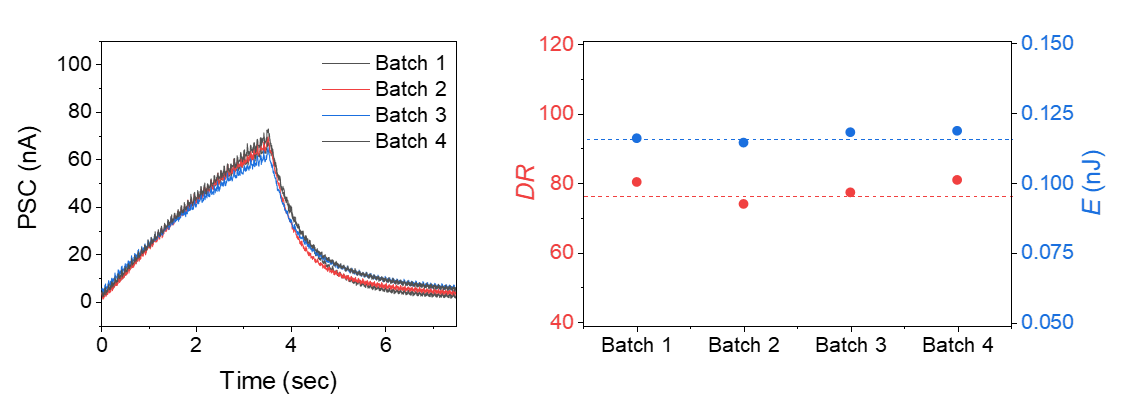


**Figure S12**. Representative LTP–LTD characteristics and the extracted *DR* and *E* values of devices from different fabrication batches, showing nearly identical conductance modulation.


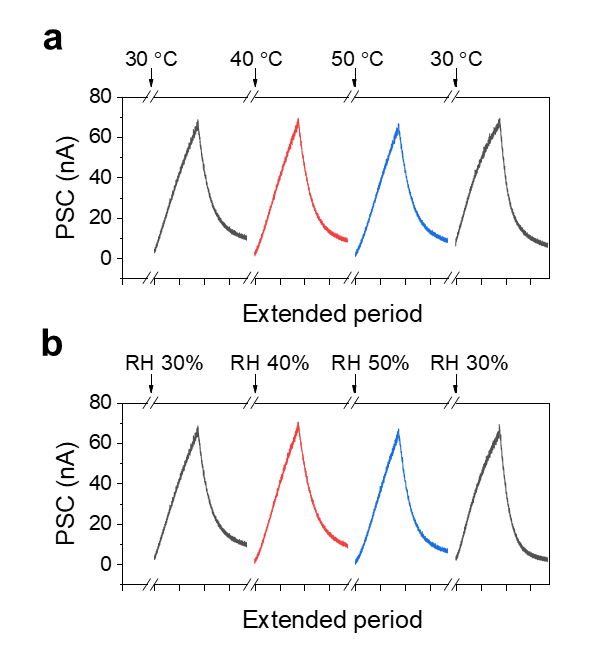


**Figure S13**. Reversible recovery of device performance after environmental cycling by altering (a) temperature at fixed RH 30% and (b) humidity at fixed 30 °C. Each break indicates the stabilization period for a given environmental condition, during which the device was kept in the environmental chamber for 5 min. For each condition, 100 pulses were applied to complete a single LTP–LTD cycle, which taking approximately 7 sec.


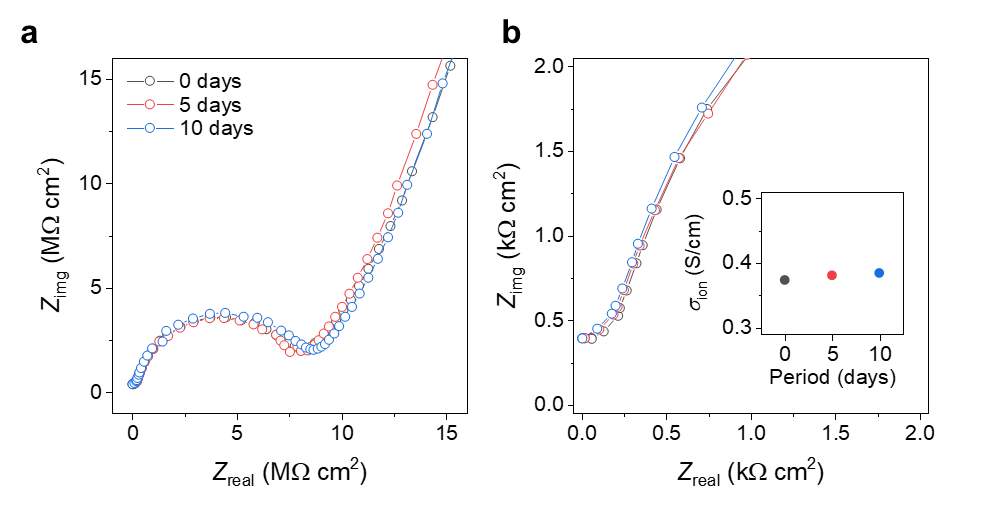


**Figure S14**. Long-term impedance characterizations of the parallel cell sandwiching solid electrolyte, noted by Nyquist plots with (a) low magnification and (b) high magnification focusing on high frequency region.

**
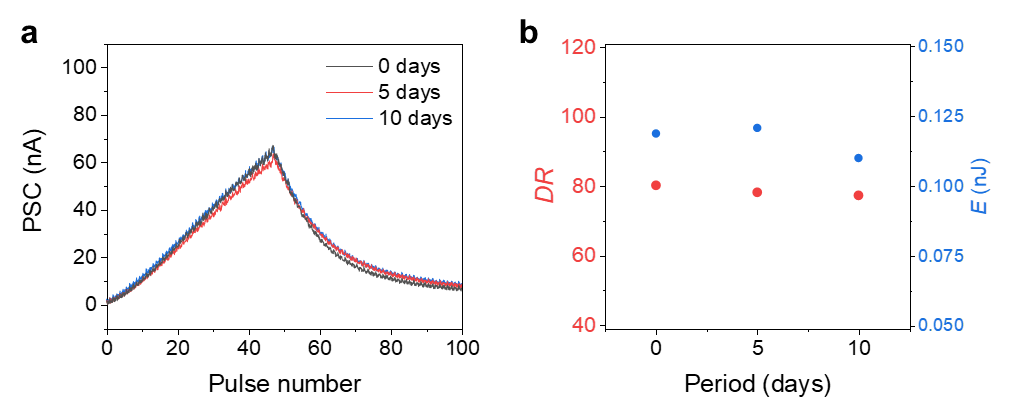
**

**Figure S15**. Long-term stability of Al NP-engineered EGMT, noted by LTP–LTD cycle of (a) pristine (0 days) and (b) 10 days-extended devices. The *V*_DS_ was fixed at 1 mV.


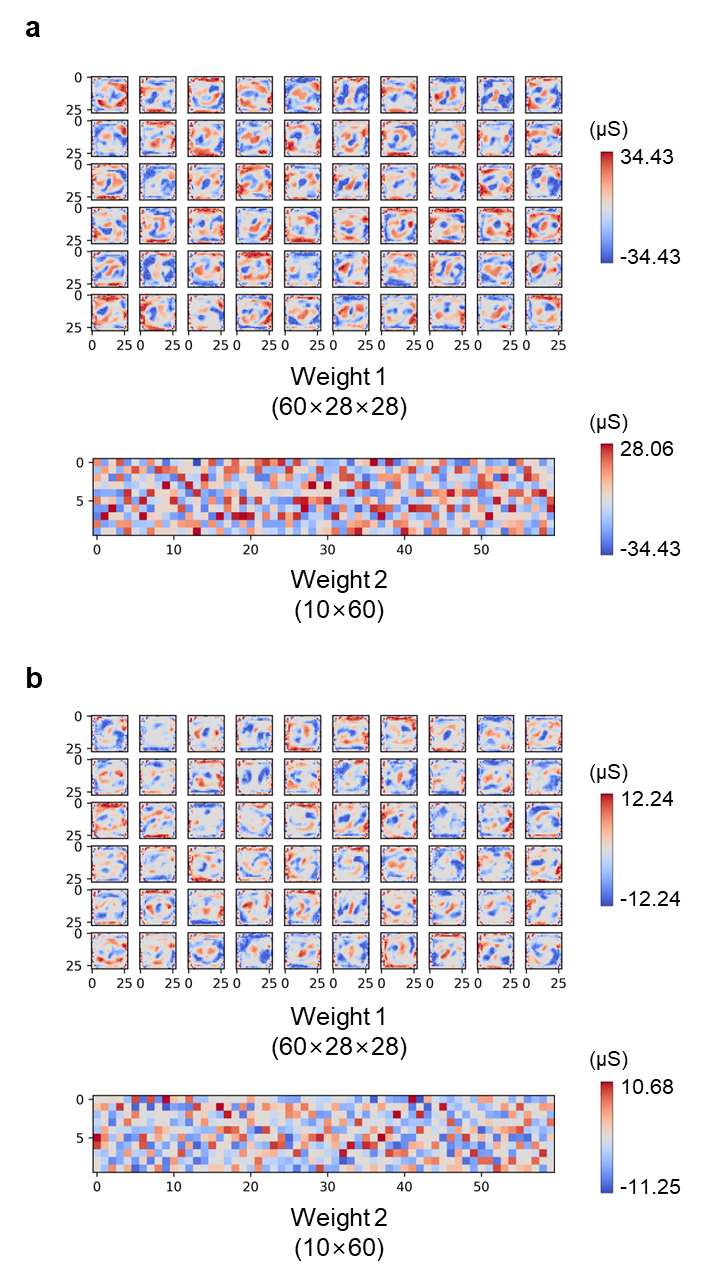


**Figure S16**. The contour plot of trained weight map (weight 1 for upper plot and weight 2 for lower plot) for the different *V*_DS_ value of a) 10 mV and b) 100 mV.


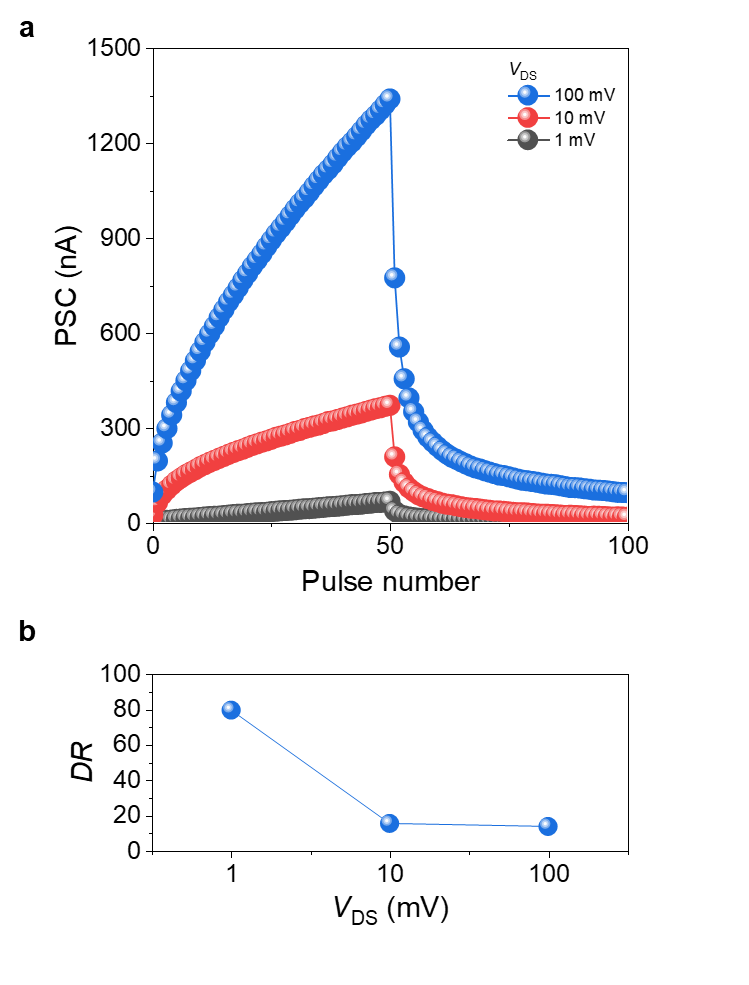


**Figure S17**. a) LTP and LTD characteristics with program/erase pulses under various *V*_DS_ (100, 10, and 1 mV). b) Plots of *DR* as a function of *V*_DS_.


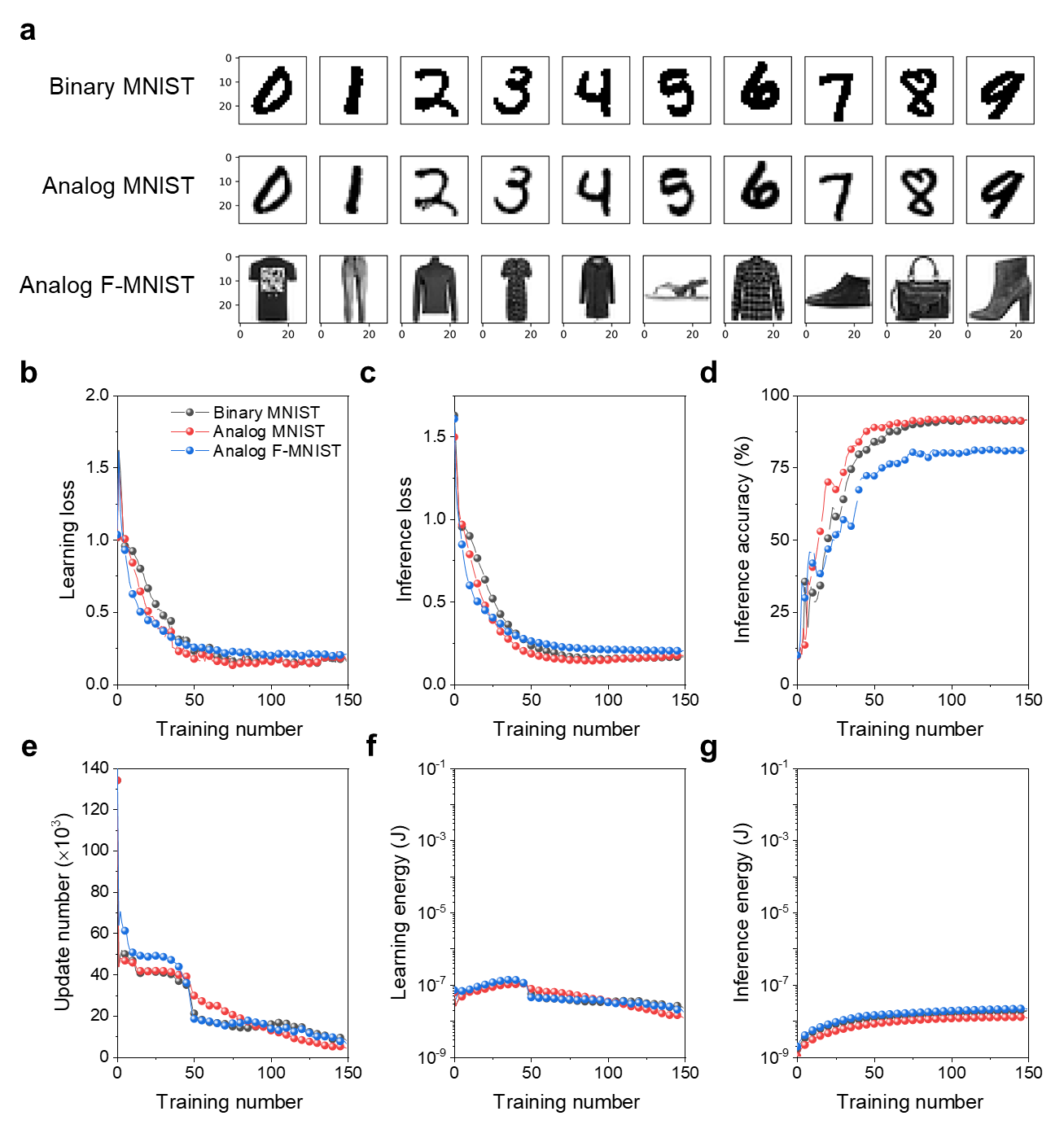


**Figure S18**. a) Representative images in different datasets of binary MNIST, analog MNIST, and analog Fashion-MNIST (F-MNIST). Neural network perceptron performances as b) learning loss value for 500 learning images which are different for each training epoch, c) inference loss value for 10,000 inference images, and d) inference accuracy for 10,000 inference images for different datasets. Energy consumption analysis as e) weight update number, f) learning energy, and g) inference energy for one 28 × 28 image processing during training process for different datasets.

**
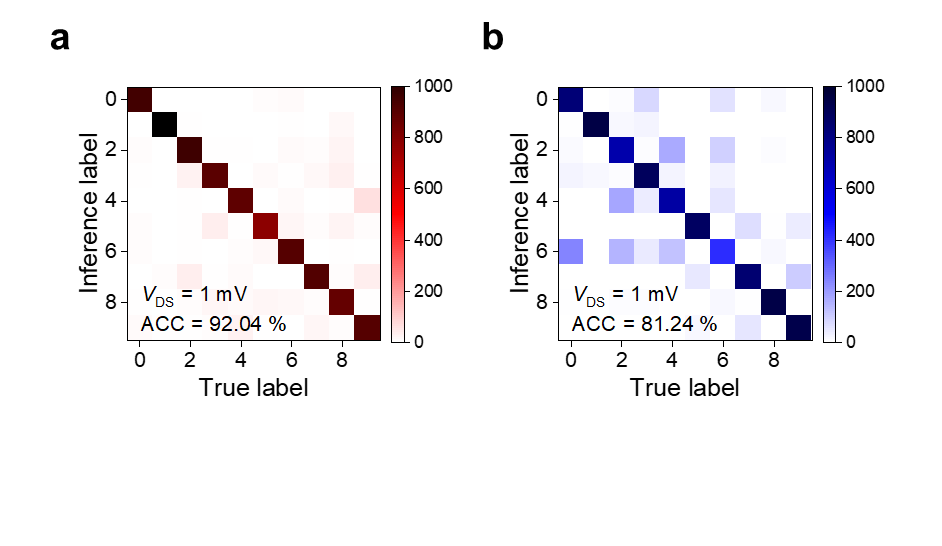
**

**Figure S19**. Confusion matrices for 10,000 inference images for the different datasets of a) analog MNIST and b) analog F-MNIST, where the row indicates true label and column indicates inferred label among 10 output classes.

**
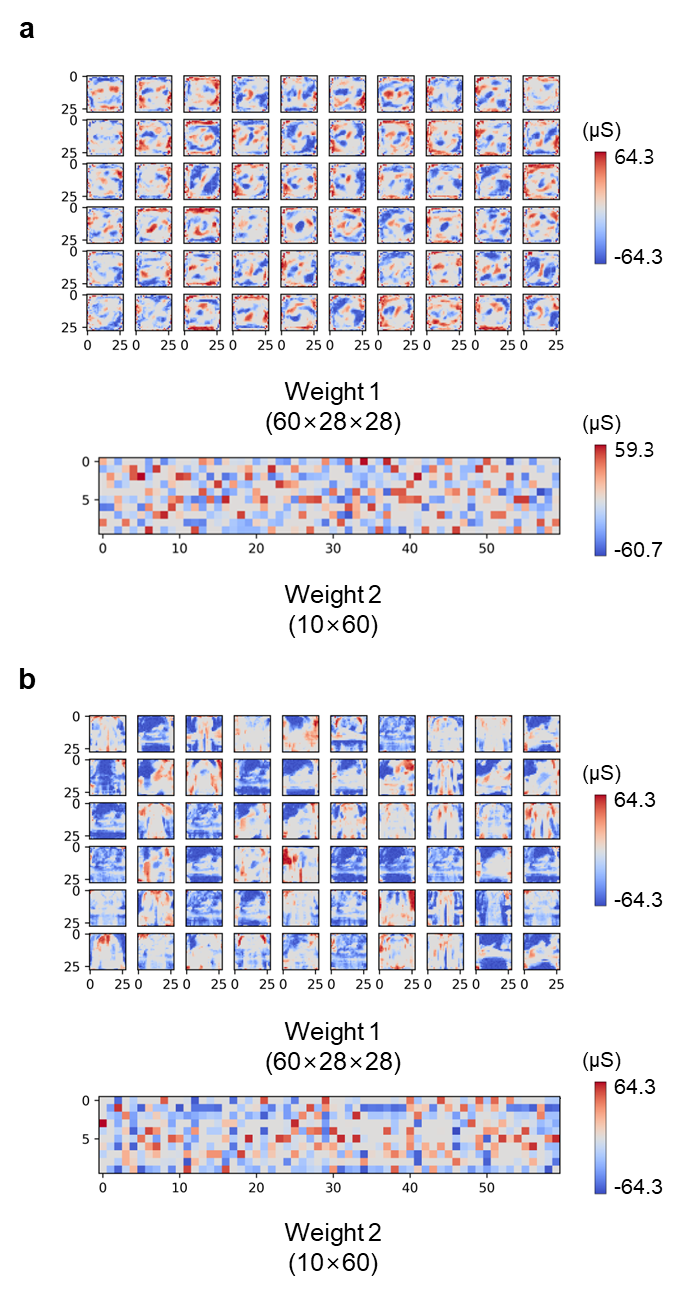
**

**Figure S20**. The contour plot of trained weight map (weight 1 for upper plot and weight 2 for lower plot) for the different datasets of a) analog MNIST and b) analog F-MNIST.

| Ref. | Type | Channel material | Dielectric  material | Pulse condition | | | *DR* | PSC  [A] | Energy consumption [J/pulse] |
| --- | --- | --- | --- | --- | --- | --- | --- | --- | --- |
|  |  |  |  | *V*_pulse_  [V] | *t*_duration_  [ms] | *V*_DS_  [V] |  |  |  |
| 1 | Oxide | α-MoO_3_ | EMIM–TFSI | 2.5 | 1 | 0.05 | 1.4 | 3.9 × 10^-9^ | 1.9 × 10^-11^ |
| 2 |  | α-MoO_3_ | PEO/LiClO_4_ | 2.5 | 10 | 0.05 | 1.7 | 3.6 × 10^-9^ | 1.8 × 10^-12^ |
| 3 |  | α-Nb_2_O_5_ | Li_x_SiO_2_ | 3 | 0.5 | - | 2.2 | - | 2.0 × 10^-12^ |
| 4 |  | IGZO | Chitosan | 2 | 25 | 0.5 | - | 3.0 × 10^-8^ | 1.0 × 10^-9^ |
| 5 |  | IGZO | Bio-polysaccharide | 0.5 | 10 | 0.5 | - | 2.7 × 10^-8^ | 1.4 × 10^-10^ |
| 6 |  | IGZO | LATP | -5 | 10 | 1 | 1.4 | 2.7 × 10^-7^ | 2.7 × 10^-9^ |
| 7 |  | IGZO | LiPON/LiSiOx | -3 | 700 | 3 | 1.7 | 1.1 × 10^-9^ | 2.4 × 10^-9^ |
| 8 |  | IGZO | EMIM-TFSI | -1 | 50 | 0.1 | 21.7 | 2.2 × 10^-9^ | 1.1 × 10^-11^ |
| 9 |  | IGZO | EMIM-TFSI | - | - | 0.1 | 10.7 | - | 3.5 × 10^-12^ |
| 10 |  | IGZO | PVA/Chitosan | 1 | 10 | 1 | 6.3 | 1.0 × 10^-8^ | 1.0 × 10^-10^ |
| 11 |  | IGZO | PSG | 1 | 100 | 1 | 3.6 | 2.6 × 10^-8^ | 2.6 × 10^-9^ |
| 12 |  | IGZO | Chitosan | 1.4 | 25 | 1 | - | 3.1 × 10^-9^ | 7.8 × 10^-11^ |
| 13 |  | In_2_O_3_ | PEO/LiClO_4_ | 0.5 | 10 | 0.5 | 3.5 | 1.4 × 10^-7^ | 7.0 × 10^-10^ |
| 14 |  | In_2_O_3_ | Lactose–citrate acid | 1 | 5 | 0.3 | 4.8 | 5.0 × 10^-7^ | 7.5 × 10^-10^ |
| 15 |  | In_2_O_3_ | PEO/LiClO_4_ | -3 | 50 | 2 | 15.0 | 1.7 × 10^-8^ | 1.7 × 10^-9^ |
| 16 |  | Li:InO_x_ | Li:AlO_x_ | 3 | 500 | 0.1 | 67 | 4.6 × 10^-7^ | 2.3 × 10^-8^ |
| 17 |  | ITO | Chitosan | 1.5 | 10 | 0.05 | 2.5 | 4.5 × 10^-6^ | 6.0 × 10^-8^ |
| 18 |  | ITO | MAE | 1 | 10 | 0.1 | 2.2 | 3.6 × 10^-5^ | 3.6 × 10^-8^ |
| 19 |  | IZO | Nano-granular SiO_2_ | 0.3 | 10 | 0.5 | - | 1.3 × 10^-8^ | 6.5 × 10^-11^ |
| 20 |  | IZO | GO | 0.1 | 10 | 0.1 | - | 5.0 × 10^-9^ | 5.0 × 10^-12^ |
| 21 |  | IZO | Albumen | 1 | 20 | 2.5 | - | 4.0 × 10^-8^ | 2.0 × 10^-9^ |
| 22 |  | IZO | Nano-granular SiO_2_ | 0.5 | 20 | 0.5 | - | 3.1 × 10^-8^ | 3.1 × 10^-10^ |
| 23 |  | IZO | Chitosan | 0.1 | 10 | 0.07 | - | 5.6 × 10^-9^ | 3.9 × 10^-12^ |
| 24 |  | Mg:SnO_2_ | PEO/LiClO_4_ | 1 | 10 | 1 | 2.5 | 3.0 × 10^-7^ | 3.0 × 10^-9^ |
| 25 |  | PFOTS_5_/ZnO | Li^+^ electrolyte | 3 | 50 | 0.1 | 14.1 | 4.4 × 10^-6^ | 2.2 × 10^-8^ |
| 26 |  | SIZO | EMIM-TFSI | 5 | 50 | 1 | 44.8 | - | 9.0 × 10^-11^ |
| 27 |  | SrFeOx | DEME-TFSI | -1.8 | 100 | 0.04 | 1.7 | 1.2 × 10^-9^ | 4.8 × 10^-12^ |
| 28 |  | WO_2.7_ | Li_3_PO_4_ | 3 | 1000 | 0.1 | 6.4 | 3.0 × 10^-6^ | 1.4 × 10^-4^ |
| 29 |  | WO_3_ | DEME-TFSI | 0.6 | 70 | 0.3 | 29 | 1.7 × 10^-9^ | 3.6 × 10^-11^ |
| 30 |  | WO_3_ | PVA/LiClO_4_ | 3 | 100 | 0.5 | 28 | 9.0 × 10^-9^ | 4.5 × 10^-10^ |
| 31 | LDM | MoS_2_ | DEME-TFSI | 2 | 10.5 | 0.05 | 12 | 4.6 × 10^-9^ | 4.8 × 10^-12^ |
| 32 |  | MoS_2_ | PVA | 1 | 10 | 0.1 |  | 2.4 × 10^-9^ | 2.4 × 10^-11^ |
| 33 |  | MoS_2_ | Chitosan | 1.5 | 10 | 0.1 | 4 | 1.0 × 10^-8^ | 5.0 × 10^-12^ |
| 34 |  | MoS_2_ | PEO:LiClO_4_ | 1 | 50 | 0.1 | >100 | 1.0 × 10^-7^ | 5.0 × 10^-10^ |
| 35 |  | CsPbBr_3_ QDs/MoS_2_ | Triflate | 0.3 | 10 | 0.1 | 3 | 5.2 × 10^-7^ | 4.3 × 10^-8^ |
| This  Work | | IGZO | PEO/LiAsF_6_ | 1 | 50 | 0.001 | 78 | 1.23×10^-8^ | 6.2 × 10^-13^ |

**Table S1**. Benchmarking table of the literature survey of the selected materials based neuromorphic devices. *V*_pulse_, gate-pulse volage; *t*_duration_, pulse duration time; *V*_DS_, drain voltage; PSC, postsynaptic current; LDM, low-dimensional materials; PFOTS_5_/ZnO, 1H,1H,2H,2H-perfluorooctyltriethoxysilane-treated zinc oxide; EMIM-TFSI, 1-ethyl-3-methylimidazolium bis(trifluoromethylsulfonyl)imide; PEO, polyethylene oxide; LATP, lithium aluminum titanium phosphate; PVA, poly(vinyl alcohol); PSG, phosphosilicate glass; MAE, maltose–ascorbic acid electrolyte; GO, graphene oxide; DEME-TFSI, diethyl methyl(2-methoxyethyl)ammonium bis(trifluoromethyl sulfonyl)imide).

| Al NP thickness [nm] | 0 | | 2 | | 4 | | 6 | |
| --- | --- | --- | --- | --- | --- | --- | --- | --- |
|  | Fitted value | Error | Fitted value | Error | Fitted value | Error | Fitted value | Error |
| *R*_s_ [Ω cm^2^] | 85 | 0.95% | 150 | 0.98% | 139 | 0.91% | 139 | 0.92% |
| CPE-T [F/cm^2^] (Q) | 1.57 × 10^-9^ | 3.51% | 2.90 × 10^-9^ | 1.78% | 2.14 × 10^-9^ | 1.72% | 1.80 × 10^-9^ | 1.89% |
| CPE-P (α) | 0.94 | 1.02% | 0.92 | 1.02% | 0.92 | 1.07% | 0.93 | 1.02% |
| *R*_ct_ [Ω cm^2^] | 6924 | 1.69% | 7024 | 2.14% | 7219 | 1.49% | 9150 | 1.69% |
| *W*_s_(*R*) [Ω cm^2^] | 2.30 × 10^7^ | 1.12% | 2.29 × 10^7^ | 2.39% | 2.30 × 10^7^ | 1.12% | 2.30 × 10^7^ | 1.12% |
| *W*_s_(*T*) [F] | 18.783 | 2.77% | 18.202 | 2.97% | 18.047 | 3.43% | 16.228 | 5.95% |
| *W*_s_(*P*) | 0.79481 | 0.91% | 0.81 | 1.06% | 0.81873 | 0.52% | 0.79481 | 0.91% |

**Table S2**. Extracted fitting parameters from EIS analysis of device cells with different NP thicknesses. The individual errors ensure the reliability of fitting.

| Al NP thickness [nm] | 0 | 2 | 4 | 6 |
| --- | --- | --- | --- | --- |
| *C*_int_ [nF/cm^2^] | 0.755 | 1.13 | 0.817 | 0.787 |
| *R*_diff_ [Ω cm^2^] | 2.30 × 10^7^ | 2.29 × 10^7^ | 2.30 × 10^7^ | 2.30 × 10^7^ |

**Table S3**. Calculated interfacial capacitance (*C*_int_) and ion diffusion resistance (*R*_diff_) obtained from the fitting parameters in Table S2 using the Brug model.

| Temperature  [°C] | *DR* | | | *E* [nJ] | | |
| --- | --- | --- | --- | --- | --- | --- |
|  | RH 30% | RH 40% | RH 50% | RH 30% | RH 40% | RH 50% |
| 30 | 76.2 | 78.3 | 74.6 | 0.116 | 0.113 | 0.101 |
| 40 | 76.9 | 70.7 | 71.1 | 0.112 | 0.100 | 0.090 |
| 50 | 74.0 | 72.1 | 66.4 | 0.108 | 0.105 | 0.084 |

**Table S4**. Summary of average *DR* and *E* of NP-engineered EGMTs under varying temperature and relative humidity.

| Type of dataset | Learning energy | | Inference energy | |
| --- | --- | --- | --- | --- |
|  | 32-bit CMOS | EGMT | 32-bit CMOS | EGMT |
| Binary MNIST | 2.28 mJ | 7.67 μJ | 22.9 μJ | 1.98 μJ |
| Analog MNIST | 2.31 mJ | 7.85 μJ | 22.9 μJ | 1.37 μJ |
| Analog F-MNIST | 2.47 mJ | 8.39 μJ | 22.9 μJ | 2.31 μJ |

**Table S5**. Comparison of learning energy and inference energy after the 150 training epochs between 32-bit CMOS and Al NP-engineered EGMT for different datasets as binary MNIST, analog MNIST, and analog F-MNIST, where the *V*_DS_ = 1 mV.

| Ref. | Memory structure | Writing energy [pJ/bit] | Learning energy during 150 epochs [μJ] |
| --- | --- | --- | --- |
| 36 | 45 nm CMOS | 20 | 2280 |
| 37 | 40 nm CMOS (HD-CIM:MRAM/SRAM) | 0.242 | 27.5 |
| 38 | STT-MTJ | 2.2 | 250 |
| 39 | 65 nm CMOS | 6 | 683 |
| 40 | Hf_0.5_Zr_0.5_O_2_ FeRAM | 0.7 | 79.6 |
| 41 | 40 nm CMOS | 10 | 1140 |
| Our work | EGMT | - | 7.67 |

**Table S6**. Comparison of learning energy after the 150 training epochs for various CMOS memory technologies and Al NP-engineered EGMT. HD-CIM, hybrid-device computing-in-memory; MRAM, magnetic random-access memory; SRAM, static random-access memory; STT-MTJ, spin-transfer torque-magnetic tunnel junction; FeRAM, ferroelectric random-access memory.

| Ref. | VMM module structure | Multiplication energy[pJ/op] | Inference energy during 150 epochs [μJ] |
| --- | --- | --- | --- |
| 36 | 45 nm CMOS | 3.2 | 22.9 |
| 42 | 65 nm CMOS (radix-4) | 16.2 | 116 |
| 43 | 16 nm FinFET (radix-4) | 1.54 | 11.0 |
| 44 | 16 nm FinFET (bit fusion) | 13.18 | 94.2 |
| 45 | 16 nm FinFET (Wallace-tree) | 2.69 | 19.2 |
| 46 | 40 nm CMOS (radix-4) | 1.56 | 11.1 |
| Our work | EGMT | - | 1.98 |

**Table S7**. Comparison of inference energy after the 150 training epochs for various CMOS VMM technologies and Al NP-engineered EGMT.

**References**

1. Yang CS, Shang DS, Liu N, et al. A Synaptic Transistor based on Quasi‐2D Molybdenum Oxide. *Advanced Materials*. 2017;29(27):1700906.

2. Yang CS, Shang DS, Liu N, et al. All-Solid-State Synaptic Transistor with Ultralow Conductance for Neuromorphic Computing. *Advanced Functional Materials*. 2018;28(42):1804170.

3. Xu H, Fang R, Wu S, et al. Li‐Ion‐Based Electrolyte‐Gated Transistors with Short Write‐Read Delay for Neuromorphic Computing. *Adv Elect Materials*. 2023;9(2):2200915.

4. He Y, Nie S, Liu R, Jiang S, Shi Y, Wan Q. Spatiotemporal Information Processing Emulated by Multiterminal Neuro‐Transistor Networks. *Advanced Materials*. 2019;31(21):1900903.

5. Guo YB, Zhu LQ, Long TY, Wan DY, Ren ZY. Bio-polysaccharide electrolyte gated photoelectric synergic coupled oxide neuromorphic transistor with Pavlovian activities. *J Mater Chem C*. 2020;8(8):2780-2789.

6. Park JM, Hwang H, Song MS, et al. All-Solid-State Synaptic Transistors with Lithium-Ion-Based Electrolytes for Linear Weight Mapping and Update in Neuromorphic Computing Systems. *ACS Appl Mater Interfaces*. 2023;15(40):47229-47237.

7. Kim S, Choi S, Kim M, Yun K, Wang G, Seong T. Dual‐Electrolyte Neuromorphic Transistor for Risk Detection and Image Processing. *Adv Materials Technologies*. Published online November 30, 2024:2401617.

8. Sun J, Oh S, Choi Y, et al. Optoelectronic Synapse Based on IGZO‐Alkylated Graphene Oxide Hybrid Structure. *Adv Funct Materials*. 2018;28(47):1804397.

9. Kwon SM, Kang SH, Cho SS, et al. Bidirectionally Modulated Synaptic Plasticity with Optically Tunable Ionic Electrolyte Transistors. *ACS Appl Electron Mater*. 2022;4(6):2629-2635.

10. Lee DH, Park H, Cho WJ. Synaptic Transistors Based on PVA: Chitosan Biopolymer Blended Electric-Double-Layer with High Ionic Conductivity. *Polymers*. 2023;15(4):896.

11. Mah DG, Park H, Cho WJ. Synaptic Plasticity Modulation of Neuromorphic Transistors through Phosphorus Concentration in Phosphosilicate Glass Electrolyte Gate. *Nanomaterials*. 2024;14(2):203.

12. He Y, Nie S, Liu R, Shi Y, Wan Q. Indium–Gallium–Zinc–Oxide Schottky Synaptic Transistors for Silent Synapse Conversion Emulation. *IEEE Electron Device Lett*. 2019;40(1):139-142.

13. Zhu Y, Liu G, Xin Z, Fu C, Wan Q, Shan F. Solution-Processed, Electrolyte-Gated In_2_ O_3_ Flexible Synaptic Transistors for Brain-Inspired Neuromorphic Applications. *ACS Appl Mater Interfaces*. 2020;12(1):1061-1068.

14. Qiu H, Hao D, Li H, et al. Transparent and biocompatible In2O3 artificial synapses with lactose–citric acid electrolyte for neuromorphic computing. *Applied Physics Letters*. 2022;121(18):183301.

15. Liu C, Shen X, Fan S, Xu T, Zhang J, Su J. Synaptic Characteristics and Neuromorphic Computing Enabled by Oxygen Vacancy Migration Based on Porous In_2_ O_3_ Electrolyte-Gated Transistors. *ACS Appl Electron Mater*. 2023;5(8):4657-4666.

16. Wang Q, Zhao T, Zhao C, et al. Solid‐State Electrolyte Gate Transistor with Ion Doping for Biosignal Classification of Neuromorphic Computing. *Adv Elect Materials*. 2022;8(7):2101260.

17. Yu F, Zhu LQ, Xiao H, Gao WT, Guo YB. Restickable Oxide Neuromorphic Transistors with Spike‐Timing‐Dependent Plasticity and Pavlovian Associative Learning Activities. *Adv Funct Materials*. 2018;28(44):1804025.

18. Qin W, Kang BH, Kim HJ. Flexible Artificial Synapses with a Biocompatible Maltose–Ascorbic Acid Electrolyte Gate for Neuromorphic Computing. *ACS Appl Mater Interfaces*. 2021;13(29):34597-34604.

19. Zhu LQ, Wan CJ, Guo LQ, Shi Y, Wan Q. Artificial synapse network on inorganic proton conductor for neuromorphic systems. *Nat Commun*. 2014;5(1):3158.

20. Wan CJ, Zhu LQ, Liu YH, et al. Proton‐Conducting Graphene Oxide‐Coupled Neuron Transistors for Brain‐Inspired Cognitive Systems. *Advanced Materials*. 2016;28(18):3557-3563.

21. Wu G, Feng P, Wan X, Zhu L, Shi Y, Wan Q. Artificial Synaptic Devices Based on Natural Chicken Albumen Coupled Electric-Double-Layer Transistors. *Sci Rep*. 2016;6(1):23578.

22. Zhou J, Wan C, Zhu L, Shi Y, Wan Q. Synaptic Behaviors Mimicked in Flexible Oxide-Based Transistors on Plastic Substrates. *IEEE Electron Device Lett*. 2013;34(11):1433-1435.

23. Liu YH, Zhu LQ, Feng P, Shi Y, Wan Q. Freestanding Artificial Synapses Based on Laterally Proton‐Coupled Transistors on Chitosan Membranes. *Advanced Materials*. 2015;27(37):5599-5604.

24. Chen B, Sun S, Fan S, Liu X, Li Q, Su J. Low‐Cost Fabricated MgSnO Electrolyte‐Gated Synaptic Transistor with Dual Modulation of Excitation and Inhibition. *Adv Elect Materials*. 2022;8(12):2200864.

25. Jin M, Lee H, Im C, et al. Interfacial Ion‐Trapping Electrolyte‐Gated Transistors for High‐Fidelity Neuromorphic Computing. *Adv Funct Materials*. 2022;32(24):2201048.

26. Oh S, Cho JI, Lee BH, et al. Flexible artificial Si-In-Zn-O/ion gel synapse and its application to sensory-neuromorphic system for sign language translation. *Sci Adv*. 2021;7(44):eabg9450.

27. Ge C, Liu C, Zhou Q, et al. A Ferrite Synaptic Transistor with Topotactic Transformation. *Advanced Materials*. 2019;31(19):1900379.

28. Lee J, Nikam RD, Lim S, Kwak M, Hwang H. Excellent synaptic behavior of lithium-based nano-ionic transistor based on optimal WO_2.7_ stoichiometry with high ion diffusivity. *Nanotechnology*. 2020;31(23):235203.

29. Yang J, Ge C, Du J, et al. Artificial Synapses Emulated by an Electrolyte‐Gated Tungsten‐Oxide Transistor. *Advanced Materials*. 2018;30(34):1801548.

30. Nikam RD, Kwak M, Lee J, Rajput KG, Hwang H. Controlled Ionic Tunneling in Lithium Nanoionic Synaptic Transistor through Atomically Thin Graphene Layer for Neuromorphic Computing. *Adv Elect Materials*. 2020;6(2):1901100.

31. John RA, Liu F, Chien NA, et al. Synergistic Gating of Electro-Iono-Photoactive 2D Chalcogenide Neuristors: Coexistence of Hebbian and Homeostatic Synaptic Metaplasticity. *Advanced Materials*. 2018;30(25):1800220.

32. Jiang J, Guo J, Wan X, et al. 2D MoS2 Neuromorphic Devices for Brain-Like Computational Systems. *Small*. 2017;13(29):1700933.

33. Hu W, Jiang J, Xie D, Liu B, Yang J, He J. Proton–electron-coupled MoS2 synaptic transistors with a natural renewable biopolymer neurotransmitter for brain-inspired neuromorphic learning. *J Mater Chem C*. 2019;7(3):682-691.

34. Bao L, Zhu J, Yu Z, et al. Dual-Gated MoS2 Neuristor for Neuromorphic Computing. *ACS Appl Mater Interfaces*. 2019;11(44):41482-41489.

35. Cheng Y, Li H, Liu B, et al. Vertical 0D-Perovskite/2D-MoS2 van der Waals Heterojunction Phototransistor for Emulating Photoelectric-Synergistically Classical Pavlovian Conditioning and Neural Coding Dynamics. *Small*. 2020;16(45):2005217.

36. Horowitz M. 1.1 Computing’s energy problem (and what we can do about it). In: *2014 IEEE International Solid-State Circuits Conference Digest of Technical Papers (ISSCC).* 2014:10-14.

37. Zhang H et al. HD-CIM: Hybrid-Device Computing-In-Memory Structure Based on MRAM and SRAM to Reduce Weight Loading Energy of Neural Networks. In *IEEE Transactions on Circuits and Systems I: Regular Papers*. vol. 69, no. 11, pp. 4465-4474, Nov. 2022.

38. Monga K, Chaturvedi N, Gurunarayanan S. Design of a STT-MTJ Based Random-Access Memory With In-situ Processing for Data-Intensive Applications. In *IEEE Transactions on Nanotechnology*. vol. 21, pp. 455-465, 2022.

39. Bose S K, Singla D, Basu A. A 51.3-TOPS/W, 134.4-GOPS In-Memory Binary Image Filtering in 65-nm CMOS. In *IEEE Journal of Solid-State Circuits*. vol. 57, no. 1, pp. 323-335, Jan. 2022.

40. Jiang P et al. A 256 Kbit Hf0.5Zr0.5O2-based FeRAM Chip with Scaled Film Thickness (sub-8nm), Low Thermal Budget (350oC), 100% Initial Chip Yield, Low Power Consumption (0.7 pJ/bit at 2V write voltage), and Prominent Endurance (>1012). *2023 International Electron Devices Meeting (IEDM)*. San Francisco, CA, USA, 2023, pp. 1-4.

41. Upton L R et al. EMBER: A 100 MHz, 0.86 mm2, Multiple-Bits-per-Cell RRAM Macro in 40 nm CMOS with Compact Peripherals and 1.0 pJ/bit Read Circuitry. *ESSCIRC 2023- IEEE 49th European Solid State Circuits Conference (ESSCIRC)*. Lisbon, Portugal, 2023, pp. 469-472.

42. Rocha L M G, Macedo M, Paim G, Costa E, Bampi S. Improving the Partial Product Tree Compression on Signed Radix-2m Parallel Multipliers. *2020 18th IEEE International New Circuits and Systems Conference (NEWCAS)*. Montreal, QC, Canada, 2020, pp. 182-185.

43. Shavit N, Stanger I, Taco R, Lanuzza M, Fish A. A 0.8-V, 1.54-pJ/940-MHz Dual-Mode Logic-Based 16×16-b Booth Multiplier in 16-nm FinFET. in *IEEE Solid-State Circuits Letters*. vol. 3, pp. 314-317, 2020.

44. Stanger I et al. FlexDML: High Utilization Configurable Multimode Arithmetic Units Featuring Dual Mode Logic. in *IEEE Solid-State Circuits Letters*. vol. 6, pp. 73-76, 2023.

45. Shavit N, Stanger I, Taco R, Fish A, Levi I. Programmable All-in-One 4×8-/2×16-/1×32-Bits Dual Mode Logic Multiplier in 16 nm FinFET With Semi-Automatic Flow. in *IEEE Access*. vol. 11, pp. 116206-116218, 2023.

46. Zhu X, Li H, Song Y, Chen Y, Guo X. High Energy Efficiency Radix-4 Booth Multiplier with Zero Encoding Skipping Mechanism. 2024 *IEEE Computer Society Annual Symposium on VLSI (ISVLSI)*. Knoxville, TN, USA, 2024, pp. 228-233.
